# A Portable One-Tube Assay Integrating RT-RPA and CRISPR/Cas12a for Rapid Visual Detection of Eurasian Avian-like H1N1 Swine Influenza Virus in the Field

**DOI:** 10.3390/v18010047

**Published:** 2025-12-28

**Authors:** Changhai Tian, Lulu Feng, Xu Zhou, Kailun Huang, Feifei Wang, Ru Luo, Fei Meng, Huanliang Yang, Chuanling Qiao, Xiurong Wang, Jianzhong Shi, Yan Chen

**Affiliations:** State Key Laboratory of Animal Disease Control and Prevention, Harbin Veterinary Research Institute, Chinese Academy of Agricultural Sciences, Harbin 150069, China; 13125903469@163.com (C.T.); fll17838747599@163.com (L.F.); 82101215654@caas.cn (X.Z.); 17855024063@163.com (K.H.); 17899318118@163.com (F.W.); 18381622958@163.com (R.L.); mengfei@caas.cn (F.M.); yanghuanliang@caas.cn (H.Y.); qiaochuanling@caas.cn (C.Q.); wangxiurong@caas.cn (X.W.); shijianzhong@caas.cn (J.S.)

**Keywords:** visual detection, Eurasian Avian-like H1N1 swine influenza virus, RT-RPA, CRISPR, Cas12a

## Abstract

The widespread circulation of Eurasian avian-like H1N1 (EA H1N1) swine influenza virus poses significant zoonotic and pandemic risks worldwide. However, current diagnostic methods are difficult to deploy in the field, as they generally require specialized laboratory infrastructure and trained personnel. Here, we present a novel dual-signal detection platform that combines reverse transcription recombinase polymerase amplification (RT-RPA) with CRISPR/Cas12a technology for rapid, on-site EA H1N1 detection. We established an integrated one-tube assay by designing and optimizing RT-RPA primers targeting a conserved region of the hemagglutinin (HA) gene, together with engineered CRISPR/Cas12a guide RNAs exhibiting high specificity. The platform incorporates two complementary readout modes: real-time fluorescence monitoring and visual colorimetric detection using a smartphone. The assay shows excellent analytical specificity, with no cross-reactivity observed against other swine influenza virus subtypes or common swine pathogens, (including CSFV, PRRSV, PEDV, PCV, TGEV, and RV). The detection limit is 2 copies/μL, and the entire procedure can be completed within 30 mins using simple portable equipment. When evaluated on 86 clinical samples, the assay demonstrated 94.18% concordance with RT-qPCR. Compared with conventional diagnostic methods, this RT-RPA–CRISPR/Cas12a assay offers greater convenience and cost-effectiveness. Its strong potential for field-based rapid testing underscores promising application prospects in swine influenza surveillance and control programs.

## 1. Introduction

Swine influenza (SI) is a highly contagious respiratory disease in pigs caused by infection with influenza A virus [1]. Viral reassortment serves as a primary mechanism for generating novel influenza viruses with distinct biological characteristics, potentially leading to catastrophic human epidemics and pandemics [2]. Historically, the pandemic IAV strains of 1957, 1968, and 2009 all originated from reassortants between human and animal influenza viruses [3,4,5]. Pigs, serving as natural reservoirs for IAVs, are susceptible to avian, swine, and human influenza viruses, thus functioning as crucial “mixing vessels” for the generation of novel influenza viruses with pandemic potential [5]. The EA H1N1 SIV was first isolated in Europe in 1979 [4]. Recent studies have demonstrated that viral subtype has become the dominant strain in Chinese swine herds through continuous transmission. Furthermore, these investigations have confirmed EA H1N1 SIV’s capability for mammalian adaptation and transmission [6,7,8], underscoring its pandemic potential. Notably, many human infection cases have been reported both domestically and internationally [4,9]. The development of a rapid, sensitive, and easily deployable detection method for EA H1N1 SIV becomes critically important for their effective surveillance and management.

Conventional detection methods for swine influenza include hemagglutination and hemagglutination inhibition assays (HA/HI), enzyme-linked immunosorbent assay (ELISA), virus isolation (VI), and reverse transcription quantitative polymerase chain reaction (RT-qPCR). While VI enables the recovery of intact, viable viruses for subsequent research, its utility is limited by prolonged detection timelines, making it unsuitable for point-of-care clinical testing. HA/HI assays, despite their simplicity and rapid turnaround, suffer from limited sensitivity and are inadequate for early-stage diagnosis. ELISA and RT-qPCR have gained widespread adoption in influenza virus detection due to their operational simplicity, high specificity, and throughput capacity. However, these techniques require specialized personnel and sophisticated laboratory infrastructure, significantly limiting their application in point-of-care testing scenarios.

In recent years, isothermal nucleic acid amplification technologies have emerged as promising alternatives to PCR, undergoing rapid development. These include loop-mediated isothermal amplification, cross-priming amplification, strand displacement amplification, rolling circle amplification, nucleic acid sequence-based amplification, and recombinase polymerase amplification (RPA). Among these, RPA technology has gained particular prominence due to its high sensitivity, excellent specificity, equipment independence, and operational simplicity. These characteristics make it particularly suitable for diagnostic applications in resource-limited settings and point-of-care testing environments. Consequently, RPA has been widely adopted across various fields, including pathogen detection and genetically modified organism analysis [10,11,12].

The CRISPR/Cas system comprises clustered regularly interspaced short palindromic repeats (CRISPR) and CRISPR-associated proteins (Cas). In recent years, CRISPR/Cas-based diagnostic technologies have emerged as a significant focus of scientific interest. Beyond its applications in biological and medical research, the CRISPR/Cas system has been developed into a powerful molecular detection tool [13]. CRISPR/Cas12a, also known as Cpf1, has demonstrated remarkable potential for developing high-performance diagnostic tools [14]. In 2018, Doudna and colleagues first reported the DNA endonuclease-targeted CRISPR trans reporter (DETECTR) platform, which utilizes the trans-cleavage activity of Cas12a. This groundbreaking approach successfully detected human papillomavirus (HPV), marking a significant advancement in molecular diagnostics [15]. Subsequently, numerous researchers have developed various pathogen detection methods leveraging the unique properties of Cas12a [14,15,16].

**Figure 1:**
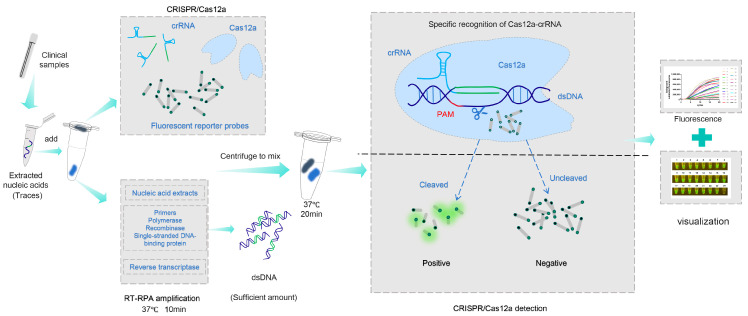
Nucleic acids extracted from clinical samples are added to the RT-RPA reaction, enabling amplification of large quantities of double-stranded DNA (dsDNA). Following centrifugation after mixing RT-RPA and CRISPR/Cas12a, the dsDNA is specifically recognized by the Cas12a-crRNA complexes. The activated Cas12a then cleaves the single-stranded DNA (ssDNA) fluorescent reporter probe. Finally, the cleaved fluorescent reporter generates detectable fluorescence signals for the identification of EA H1 SIV.

This study established a one-tube on-site visual detection platform for EA H1N1 SIV based on RT-RPA and CRISPR/Cas12a (Figure 1), and evaluated its sensitivity, specificity, and applicability for clinical sample detection.

## 2. Materials and Methods

### 2.1. Materials

The pMD-18T-EA HA plasmid and Lachnospiraceae bacterium Cas12a (LbCas12a) protein were preserved at the National Avian Influenza Reference Laboratory, Harbin Veterinary Research Institute, Chinese Academy of Agricultural Sciences. The clinical swab samples and various swine influenza virus subtypes, including Classical H1N1 (CS H1N1), 2009 pandemic H1N1 (2009/H1N1 pdm), H3, H5, H6, H9, and EA H1N1, were preserved and maintained by the National Avian Influenza Reference Laboratory at the Harbin Veterinary Research Institute of the Chinese Academy of Agricultural Sciences.

The following viral strains were provided by various research teams at Harbin Veterinary Research Institute, Chinese Academy of Agricultural Sciences, and Harbin Weike Biotechnology Development Company: Classical Swine Fever Virus (CSFV), Porcine Reproductive and Respiratory Syndrome Virus (PRRSV), Porcine Epidemic Diarrhea Virus (PEDV), Porcine Circovirus (PCV), Transmissible Gastroenteritis Virus (TGEV), and Rotavirus (RV).

### 2.2. Reagents and Instruments

The RNase inhibitor was purchased from Thermo Fisher Co., Ltd. (Nanjing, China). Fluorescent probes, CRISPR DNA (crDNA) for CRISPR RNA (crRNA) synthesis, and specific RT-RPA primers were synthesized by Beijing Ribio Biotech Co., Ltd. (Beijing, China). The RNA extraction kit was obtained from TIANGEN Biotechnology Co., Ltd. The RT-RPA kit was purchased from Genenode Biotech Co., Ltd. (Wuhan, China). The RNA purification kit was acquired from Sangon Biotech Co., Ltd. (Shanghai, China). The T7 transcription kit was obtained from New England Biolabs (Ipswich, MA, USA). NEBuffer™ r3.1 was purchased from New England Biolabs. Fluorescence signals were acquired using the QuantStudio 5 system (Thermo Fisher Scientific, Waltham, MA, USA), and the visualized results were images captured and saved by a smartphone under blue light illuminator.

### 2.3. Experimental Principle Based on RT-RPA-CRISPR/Cas12a Detection Method

We established a single-tube isothermal detection system for EA H1N1 SIV by targeting a conserved fragment of the HA gene, combining RT-RPA with CRISPR-Cas12a detection (Figure 1). Briefly, the target sequence is amplified through RT-RPA reaction, followed by detection using the CRISPR/Cas12a system, with fluorescence serving as the detection signal output.

Viral nucleic acids from clinical samples are specifically amplified by RT-RPA, converting trace amounts of RNA into substantial quantities of double-stranded DNA (dsDNA). This dsDNA is then specifically recognized by the Cas12a-crRNA complexes. Subsequently, the activated Cas12a exerts both cleavage and trans-cleavage activities, resulting in the cleavage of single-stranded DNA (ssDNA) fluorescent reporters. This cleavage separates the fluorophore and quencher groups at the ends of the reporter molecule. Consequently, the disrupted fluorescent reporter generates a detectable fluorescence signal, enabling the identification of EA H1N1 SIV. Conversely, in the absence of EA H1N1 SIV in the sample, the subsequent cascade of reactions does not occur, and no fluorescence signal is produced.

### 2.4. Preparation of Viral RNA

Viral nucleic acids from all viruses and clinical swab samples used in this study were extracted using the RNA extraction kit (TIANGEN Biotechnology Co., Ltd., Beijing, China) and stored at −80 °C for subsequent analysis.

### 2.5. Preparation of crRNA, RT-RPA Primers and Fluorescent Probes

The crRNA for LbCas12a consists of a direct repeat segment and a spacer region, with the spacer region being reverse complementary to the target DNA sequence. LbCas12a recognizes a protospacer adjacent motif (PAM) rich in thymine (T) at the 5′ end. Based on these principles, we downloaded 319 HA gene sequences of EA H1N1 SIV from the NCBI database and performed sequence alignment using MEGA11.0 to identify conserved regions. We selected a conserved fragment of approximately 21 nt in the HA gene following the PAM sequence as the target DNA, based on which we designed seven crRNAs (Table 1).

Since LbCas12a recognizes both ssDNA and dsDNA, we used the pMD-18T-EA HA plasmid as the template for Cas12a reactions to screen the seven prepared crRNAs. Based on the optimal crRNA identified through screening, we designed five pairs of RT-RPA primers to amplify the target DNA region (Table 2). The most efficient primer pair was selected as the optimal RT-RPA primer set. All fluorescent probes were synthesized by Beijing Ribio Biotech Co., Ltd. (Table 1).

### 2.6. Optimization of One-Step Reaction Conditions

The optimization of the system not only effectively reduces costs but, more importantly, maximizes the detection efficiency of the RT-RPA-CRISPR/Cas12a system. We systematically investigated and optimized various experimental conditions to achieve optimal performance. Initially, we evaluated detection efficiency by conducting RT-RPA reactions at different temperatures (37 °C and 42 °C) while maintaining other conditions constant. Subsequently, we employed a single-variable approach to optimize the RT-RPA reaction volume (2 μL, 5 μL, 10 μL, 15 μL, 20 μL) and time (5 mins, 10 mins, 15 mins, 20 mins, 25 mins, 30 mins).

Following the optimization of RT-RPA parameters, we performed gradient optimization for three key components: crRNA concentration (10 μM, 1 μM, 100 nM, 10 nM, 1 nM, 100 pM, 10 pM), LbCas12a concentration (10 μM, 1 μM, 100 nM, 10 nM, 1 nM, 100 pM, 10 pM), and fluorescent probe concentration (500 pM, 200 pM, 100 pM, 50 pM, 20 pM, 10 pM, 5 pM).

## 3. Results

### 3.1. Design and Screening of crRNA, RT-RPA Primers and Preparation of Fluorescent Probes

**Figure 2:**
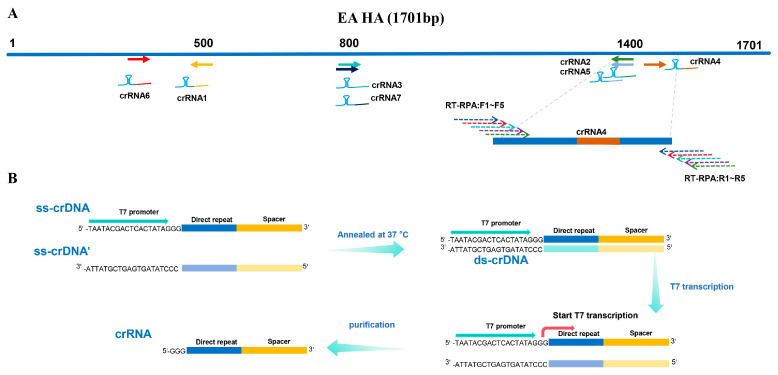
(**A**) The (EA H1 SIV)HA gene was used as a template to design crRNAs, and the RT-RPA primers for the optimal crRNA were designed. (**B**) The preparation process of crRNA involves first synthesizing the relevant crDNA and then performing T7 transcription.

We downloaded 319 HA gene sequences of EA H1N1 SIV from the NCBI database and performed sequence alignment using MEGA11.0 to identify conserved regions. A conserved fragment of approximately 21 nt in the HA gene following the PAM sequence was selected as the target DNA, based on which we designed seven crRNAs, designated as crRNA1 to crRNA7 (Table 1) (Figure 2A). The T7 promoter sequence was added to the 5′ end of the direct repeat segment, and the ss-crDNA along with its complementary sequence were synthesized. The ds-crDNA was obtained through a 12 h annealing reaction at 37 °C. Transcription was performed using the T7 transcription kit, and the products were purified using an RNA purification kit to obtain crRNA (Figure 2B). The fluorescent probe sequence, 5′-[6FAM]TTATT[BHQ1]3′, was synthesized by Beijing Ribio Biotech Co., Ltd.

**Figure 3:**
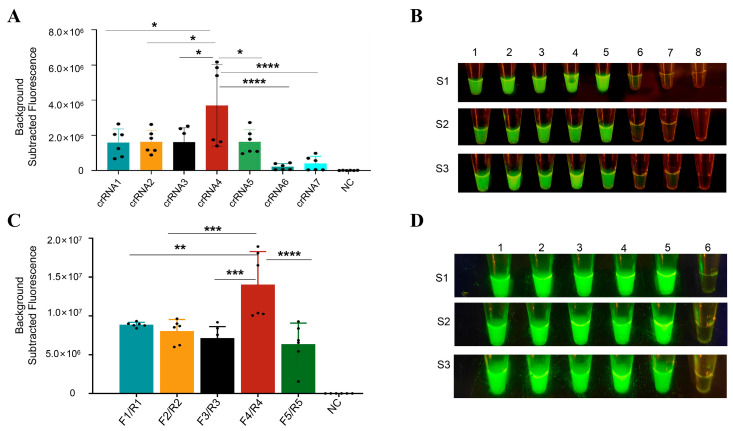
Screening of the best crRNA and RT-RPA primers. (**A**) Cleavage activity of 7 crRNA-guided LbCas12a cleavage activities of HA genes targeting EA H1 SIV. Fluorescence values of decontextualized values acquired by Quant Studio 5, data shown as average + SD (*n* = 6). (**B**) Visualization of crRNA screening results. Experiments were performed in triplicate (S1–S3), with representative images from three samples displayed. The visualization result is illuminated by blue light, and the image is captured by a smartphone. (**C**) Fluorescence values for RT-RPA primer screening, data shown as average + SD (*n* = 6). (**D**) Visualization of RT-RPA primer screening. S1–S3 are repeated experiments. Experiments were performed in triplicate (S1–S3), with representative images from three samples displayed. NC, negative control. * *p*  <  0.05; ** *p*  <  0.05; *** *p*  <  0.01; **** *p*  <  0.001.

Using the pMD-18T-EA HA plasmid as a template, Cas12a reactions were prepared with LbCas12a and the designed crRNAs (12.5 μL 1 × NEBuffer™ r3.1, 1.25 μL LbCas12a-10 μM, 1 μL crRNA-100 nM, 0.25 μL RNase inhibitors, 8 μL RNase-H_2_O, 2 μL pMD-18T-EA HA, 1 μL ssDNA probes-100 pM). After 40 mins of reaction at 37 °C, images were captured under blue light using a smartphone for screening the seven designed crRNAs. Results indicated that crRNA4 exhibited the highest fluorescence intensity and optimal visual detection (Figure 3A,B), and was, therefore, selected as the optimal crRNA.

Based on the selected optimal crRNA, five pairs of RT-RPA primers were designed (Table 2) to amplify the target region corresponding to the optimal crRNA, generating substantial amounts of target DNA. The RNA of EA H1N1 SIV was added to the RT-RPA reaction and incubated at 42 °C for 30 mins. Subsequently, 5 μL of the product was added to the CRISPR/Cas12a system and reacted at 37 °C for 40 mins in the QuantStudio 5 system. Fluorescence values were recorded, and images were captured by smartphone under blue light for RT-RPA primer screening. Results demonstrated that F4/R4 exhibited the highest fluorescence intensity and distinct visual results (Figure 3C,D) and was, therefore, selected as the optimal RT-RPA primer pair.

Consequently, crRNA4 and F4/R4 were selected for subsequent experiments.

### 3.2. Optimized RT-RPA-CRISPR/Cas12a Detection Systems

To establish an economical, rapid, and sensitive detection method while maintaining high fluorescence intensity and distinct visual results, we optimized the RT-RPA-CRISPR/Cas12a reaction conditions using a single-variable approach. We sequentially optimized the temperature, volume, and time of the RT-RPA reaction system, followed by optimization of crRNA, LbCas12a, and fluorescent probe concentrations.

**Figure 4:**
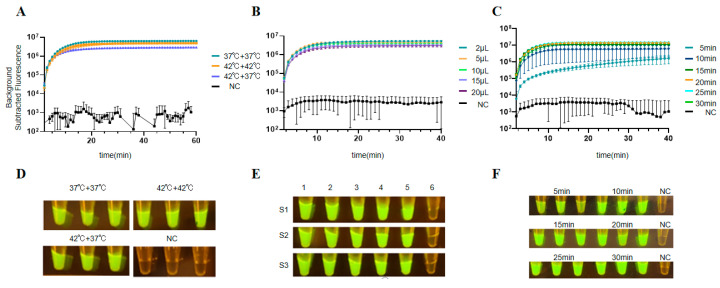
Optimization of RT-RPA reaction system. (**A**) Optimized fluorescence values for RT-RPA reaction temperature. (**B**) Optimized fluorescence value results for RT-RPA reaction volume. (**C**) Optimized fluorescence value results for RT-RPA reaction time. (**D**) Optimized visualization of RT-RPA reaction temperature. (**E**) Optimized visualization of RT-RPA reaction volumes. 1–6: 20 μL, 15 μL, 10 μL, 5 μL, 2 μL, negative control. S1–S3 are repeat groups. (**F**) Optimized visualization of RT-RPA reaction time. The reaction time of Cas12a detection is 40 mins, and the fluorescence signal is collected once per cycle. Data shown as average + SD (*n* = 3). Experiments were performed in triplicate (S1–S3), with images from three samples displayed.

**Figure 5:**
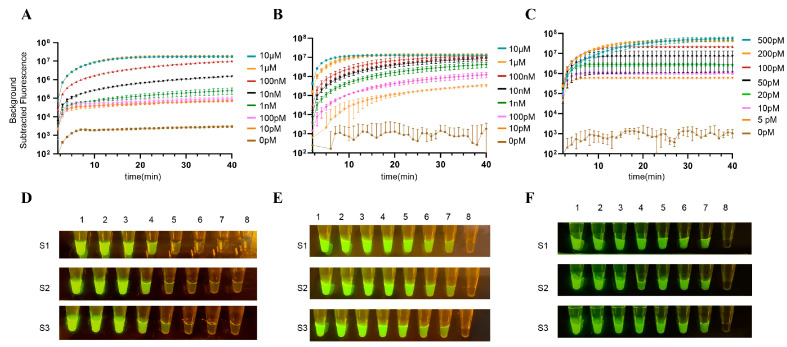
Optimization of Cas12a reaction system. (**A**) Fluorescence value results optimized for crRNA. (**B**) Fluorescence values optimized for LbCas12a. (**C**) Fluorescence value results optimized for fluorescence reporter probes. (**D**) Visualization of crRNA optimization. 1–8: 10 μM, 1 μM, 100 nM, 10 nM, 1 nM, 100 pM, 10 pM, 0 pM. (**E**) Visualization of LbCas12a optimization. 1–8: 10 μM, 1 μM, 100 nM, 10 nM, 1 nM, 100 pM, 10 pM, 0 pM. (**F**) Visualization of fluorescent reporter probes optimization. 1–8: 500 pM, 200 pM, 100 pM, 50 pM, 20 pM, 10 pM, 5 pM, 0 pM. Data shown as average + SD (*n* = 3). Experiments were performed in triplicate (S1–S3), with images from three samples displayed.

The results showed that RT-RPA combined with the CRISPR/Cas12a reaction produced higher fluorescence intensity at 37 °C (Figure 4A,D). However, when the RT-RPA reaction volume was set to 2 μL, the volume of the target nucleic acid to be detected added was 0.02 μL, which was inconvenient for practical operation. Therefore, we set the optimal RT-RPA reaction volume to 5 μL (Figure 4B,E). Considering both detection time and detection efficiency, we set the optimal RT-RPA reaction time to 10 mins (Figure 4C,F). Additionally, based on the experimental results, we determined the final reaction system composition to include crRNA (1 μM) and LbCas12a (10 μM). During the experiments, it was found that the fluorescent reporter molecule was least affected by background values when its concentration was 5 pM, so we selected a fluorescent reporter molecule concentration of 5 pM (Figure 5). The results showed that the RT-RPA-CRISPR/Cas12a system reached saturation within 20 mins after mixing, so the reaction time after mixing was set to 20 mins (Figure 4 and Figure 5).

The final optimal conditions for the RT-RPA-CRISPR/Cas12a system were established as follows: RT-RPA reaction (37 °C, 5 μL, 10 mins) and CRISPR/Cas12a reaction (crRNA-1 μM, LbCas12a-10 μM, ssDNA probes-5 pM, 20 mins). The experimental procedure was conducted as follows: First, the RT-RPA reaction mix was prepared (32.9 μL A Buffer, 2.5 μL B Buffer, 2 μL forward primer, 2 μL reverse primer). Then, 4.5 μL of the mix was combined with 0.5 μL of nucleic acid extract and placed at the bottom of the tube. Simultaneously, the Cas12a reaction mixture was prepared (12.5 μL 1 × NEBuffer™ r3.1, 1.25 μL LbCas12a, 1 μL crRNA, 0.25 μL RNase inhibitors, 8 μL RNase-H_2_O, 1 μL ssDNA probes), mixed thoroughly, and placed in the tube cap. The RT-RPA reaction proceeded at 37 °C in the dark for 10 mins. Subsequently, centrifugation was performed to thoroughly mix the Cas12a system in the tube cap with the RT-RPA system at the bottom. The mixture was then incubated at 37 °C in the QuantStudio 5 system for 20 mins, with fluorescence signals collected every minute. Upon completion, fluorescence values were recorded, and images were captured by smartphone under blue light using a smartphone.

### 3.3. Analytical Sensitivity and Specificity of CRISPR-Based EA H1N1 Detection

To evaluate the sensitivity of this method, we performed gradient dilutions of the EA H1N1 SIV viral nucleic acid, with dilution ranges from 2048 copies/μL to 1 copy/μL, and three replicates were conducted for each concentration. Additionally, 20 samples with both virus isolation and RT-qPCR results negative were used as negative controls. The nucleic acid extracts of these samples were added to the RT-RPA-CRISPR/Cas12a system, and experiments were carried out under optimized conditions.

**Figure 6:**
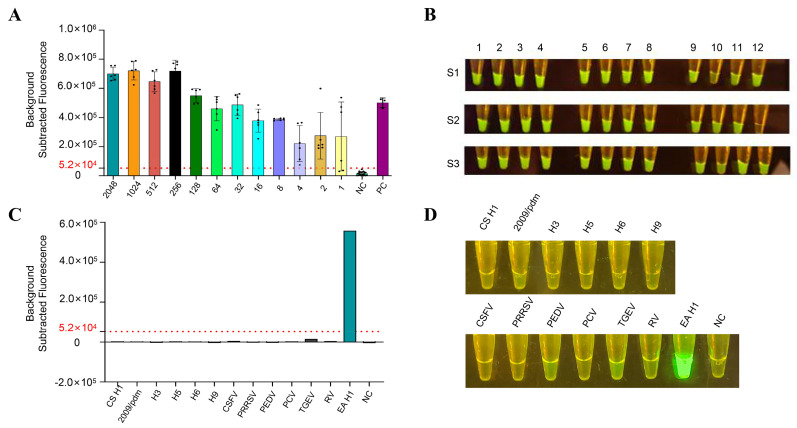
PartSensitivity experiments. (**A**) Dilute the viral nucleic acid with a concentration of 2048 copies/μL in a 2-fold serial dilution for the sensitivity detection experiment of RT-RPA-CRISPR/Cas12a, and measure the fluorescence value after 30 reactions. Data are presented as the mean ± SD, with *n* = 6 for diluted samples and *n* = 20 for the negative control (NC). (**B**) The corresponding visualization results of the sensitivity experiment. 1–12: 2048 copies/μL to 1 copy/μL. S1–S3 are repeat groups. The experiment used common pathogens in swine herds and multiple subtypes of swine influenza virus. (**C**) Fluorescence values of the specificity experiment. (**D**) Visualization results of the specificity experiment. The dashed red lines indicate the critical value of detection.

Based on the experimental results, statistical analysis was performed on the obtained data. The critical value of this detection method was defined as the mean of the 20 negative samples plus three times the standard deviation (x + 3SD = 52383.45) (Table S1). Samples with fluorescence values exceeding this value were judged as positive, and those below this value were judged as negative. The results showed that our detection method has high sensitivity, capable of detecting nucleic acids as low as 2 copies/μL, exhibiting significant fluorescence intensity (Figure 6A,B) (Table S2).

To assess the specificity of the detection method, we extracted nucleic acids from common porcine viral pathogens (CSFV, PRRSV, PEDV, PCV, TGEV, and RV) and other swine influenza virus subtypes (CS H1, 2009/pdm, H3, H5, H6, and H9). The results indicated that significant fluorescence intensity and visual detection were observed only in the EA H1N1 SIV group, while no cross-reactivity was detected with other common porcine viruses or swine influenza virus subtypes (Figure 6C,D and Figure S1 and Table S4).

Our study demonstrates that this EA H1N1 SIV detection method exhibits high sensitivity, capable of detecting nucleic acids at concentrations as low as 2 copies/μL, and excellent specificity, showing no cross-reactivity with non-influenza porcine viruses or other swine influenza virus subtypes.

### 3.4. Tests to Detect the Coincidence Rate of Clinical Swabs

**Figure 7:**
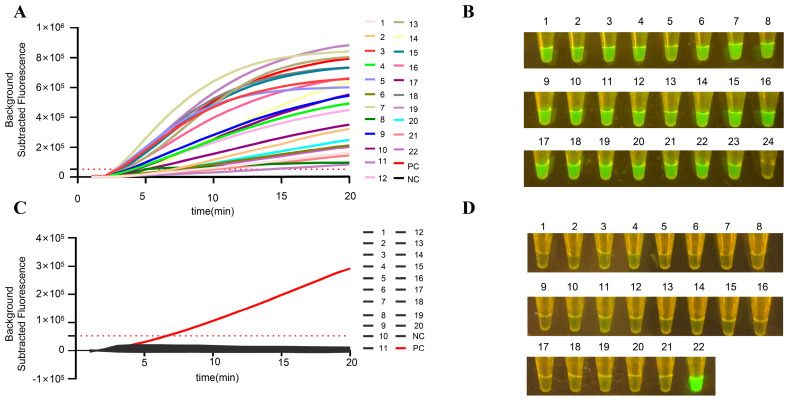
Partial detection results of RT-RPA-CRISPR/Cas12a on clinical samples. (**A**) Fluorescence values of positive samples. (**B**) The visualization results of the positive samples, 1–22 are the positive samples tested, and 23–24 are the positive control and negative control. (**C**) Visualization of the negative sample. (**D**) Fluorescence values of negative samples, 1–20 are the negative sample tested, 21–22 are the negative control and positive control. The dashed red lines indicate the critical value of detection.

To evaluate the clinical detection capability of our method, we conducted parallel testing on 86 clinical nasal swab samples (60 virus isolation-positive and 26 virus isolation-negative samples) using both commercial RT-qPCR and our RT-RPA-CRISPR/Cas12a detection method. Our results demonstrate that the developed method exhibits excellent performance in clinical sample detection. Partial detection results are presented: positive samples showed high fluorescence intensity and distinct visual results (Figure 7A,B), while negative samples displayed no fluorescence signal or visual detection (Figure 7C,D).

Furthermore, our method demonstrated high concordance with the commercial RT-qPCR assay, showing a 94.18% agreement rate compared to RT-qPCR results (Table 3 and Table S3). These findings indicate that our RT-RPA-CRISPR/Cas12a detection method is reliable for clinical application, with performance comparable to established RT-qPCR methods.

## 4. Discussion

The widespread prevalence of EA H1N1 virus in swine herds across Europe and China, coupled with its demonstrated ability to cause human infections in multiple countries and efficient transmission capacity, suggests pandemic potential for this virus [7,9]. Therefore, the development of specific, sensitive, and rapid detection methods for EA H1N1 virus is crucial for effective surveillance and control of swine influenza. In this study, we have established a single-tube detection method for EA H1N1 SIV based on RT-RPA and CRISPR/Cas12a.

The crRNA, as a crucial component, requires at least 17 or more base pair matches with the target DNA to fully activate Cas12a’s non-target cleavage activity. However, when the match is less than 20 bases, Cas12a’s trans-cleavage activity is significantly reduced [17,18]. LbCas12a recognizes a thymine (T)-rich PAM at the 5′ end. For target dsDNA, modifications in the PAM sequence or mismatches in the “seed region” adjacent to the PAM can substantially inhibit the trans-cleavage activity of ssDNA [15]. During the processes of binding, recognition, and cleavage site activation, LbCas12a is guided by crRNA and exhibits multiple conformations. The protein demonstrates highly dynamic and reversible transitions between these conformational states [17]. These findings indicate that target DNA must meet multiple stringent conditions, suggesting that crRNA designed based on target DNA is theoretically highly specific. Otherwise, LbCas12a cannot be fully activated to exert its cleavage activity. Therefore, we identified conserved regions among numerous EA H1N1 HA genes and meticulously designed seven crRNAs following the aforementioned principles (Table 1). While all seven crRNAs were theoretically expected to demonstrate efficient targeting, our experimental results revealed that crRNA6 and crRNA7 failed to achieve the desired performance. The targeting efficiency of crRNA is influenced by various factors. Studies have demonstrated that while fully complementary spacer regions ensure specific targeting by the CRISPR/Cas system, the efficiency varies depending on the length and base composition of the crRNA’s spacer region, consequently affecting detection sensitivity [19,20]. We hypothesize that the significantly low targeting efficiency of these two crRNAs may be attributed to their specific base composition. The presence of poly(T) at the 5′ end of crRNA has been associated with reduced reactivity, and when the “N” in the PAM (TTTN) is “T”, the reaction activity is notably diminished [21]. The PAM sequence corresponding to crRNA7 is TTTT. crRNA relies on folding into a specific pseudoknot structure to bind with LbCas12a, where a hairpin-like structure formed by intramolecular base pairing in the palindromic region of the crRNA repeat sequence is crucial for this pseudoknot formation. Structural changes occur when flanking sequences (upstream leader or downstream spacer) compete with nucleotides in the repeat sequence for base pairing, leading to alternative secondary structures. Such mispairing can cause crRNA to fold into structures unrecognizable by Cas12a, thereby affecting reaction efficiency [22]. We identified sequences within the spacer regions of crRNA6 and crRNA7 that could potentially base-pair with the repeat sequence. We suspect that the combination of the unique PAM (TTTT) and the potential base-pairing between the spacer and repeat sequences may contribute to their low targeting efficiency.

To meet the requirements for clinical POCT, our developed detection method must incorporate multiple advantages, including cost-effectiveness, rapid turnaround, ease of preparation, and independence from sophisticated equipment. Consequently, the result display system requires integration with appropriate and simple biosensors. Typically, colorimetric biosensors have a detection limit ranging from 10 to 100 copies/reaction [23]. However, electrochemical and microfluidic biosensors present challenges in fabrication and are cost-prohibitive. We selected a fluorescence-based biosensor, which offers visible results to the naked eye and requires only simple LED blue light equipment, making it well-suited for point-of-care testing applications. Research indicates that the catalytic efficiency of Cas effectors varies with the length of fluorescent reporter genes [24], with 10-T reporters demonstrating higher cleavage rates and detection sensitivity compared to classical reporters (TTATT) [25]. In our experiments, we substituted TTATT with this 10-T reporter, using the probe sequence 5′-[6FAM]TTTTTTTTTT[BHQ1]3′ (10T). Results showed that under identical conditions, the 10-T reporter generated stronger fluorescence signals (1.39 × 10^7^) and more distinct visual results than the classical reporter (6.5 × 10^6^). However, unexpectedly, our experiments revealed elevated background signals in negative controls, exhibiting noticeable luminescence. We investigated potential factors influencing 10-T performance, including RT-RPA components, LbCas12a, crRNA, NEBuffer™ r3.1, and intense light exposure. Results demonstrated that LbCas12a had the most significant impact on 10-T, followed by RT-RPA reactions. To ensure controlled fluorescence values and visual results, we ultimately selected the classical fluorescent reporter gene (TTATT) for subsequent experiments.

The RT-RPA reaction can provide sufficient target amplicons for the CRISPR/Cas12a system. However, conventional protocols require opening the reaction tube to transfer the amplified products into the CRISPR/Cas12a detection system [15], which significantly increases the risk of aerosol contamination and potential false-positive results. Furthermore, studies have demonstrated that Cas12a-mediated substrate cleavage competes with isothermal amplification in single-tube reactions, making it impossible to perform both reactions efficiently in the same buffer system without compromising detection performance [26]. To address these challenges, we developed an innovative single-tube detection method for EA H1N1 SIV by physically separating the two reaction systems. The RT-RPA reaction was placed at the bottom of the centrifuge tube, while the CRISPR/Cas12a reaction components were pre-loaded in the tube cap. After completing the isothermal amplification, brief centrifugation mixed the two systems for subsequent reactions. This design eliminates aerosol contamination risks associated with product transfer while allowing both the amplification reaction and Cas12a-mediated cleavage to perform optimally without compromising overall detection sensitivity.

In summary, we have successfully developed a one-tube on-site visual detection platform for EA H1N1 SIV based on RT-RPA and CRISPR/Cas12a. This novel assay demonstrates excellent specificity, high sensitivity (detection limit of 2 copies/μL), rapid turnaround time (30 mins), ease of preparation, and minimal equipment requirements. The method shows significant potential for point-of-care detection of EA H1 SIV, offering valuable applications in surveillance and control programs against EA H1N1 SIV.

## Figures and Tables

**Table 1:** crRNA and reporter probe sequences.

Name	Sequence (5′-3′)	Guide Start
crRNA1	UAAUUUCUACUAAGUGUAGAUAAUCACUGAGUUCACUUUGUU	502/reversed
crRNA2	UAAUUUCUACUAAGUGUAGAUAAUUUGAAGUCUCUAUUAUGU	1398/reversed
crRNA3	UAAUUUCUACUAAGUGUAGAUCAUUGAAAAAAGGCUCUAGUU	818/forward
crRNA4	UAAUUUCUACUAAGUGUAGAUAGUUCUAUCACAAAUGUGAUA	1448/forward
crRNA5	UAAUUUCUACUAAGUGUAGAUGAAUUUGAAGUCUCUAUUAUG	1397/reversed
crRNA6	UAAUUUCUACUAAGUGUACAUAAAGAUUUGAAAUUUUCCCAA	386/forward
crRNA7	UAAUUUCUACUAAGUGUAGAUAAGCCACUGGGAAUUUAAUAG	779/forward
report probes	[6-FAM]_TTATT_[BHQ-1]	

**Table 2:** The primer sequences used in this study.

Primer Name	Sequence (5′-3′)	Position
crRNA4-RT-RPA-F1	AACAATGCTAAGGAAATCGGAAATGGCTGC	1413
crRNA4-RT-RPA-R1	TTGTATGTGCCATTCTTTACGCTTTCCATG	1475
crRNA4-RT-RPA-F2	TAAGGAACAATGCTAAGGAAATCGGAAATG	1408
crRNA4-RT-RPA-R2	CTCCCTATTCAACTTGGATTCTTCTGAATA	1515
crRNA4-RT-RPA-F3	AACTAAGGAACAATGCTAAGGAAATCGGAA	1405
crRNA4-RT-RPA-R3	CCTCCCTATTCAACTTGGATTCTTCTGAAT	1516
crRNA4-RT-RPA-F4	CAACTAAGGAACAATGCTAAGGAAATCGGA	1404
crRNA4-RT-RPA-R4	TCCTCCCTATTCAACTTGGATTCTTCTGAA	1517
crRNA4-RT-RPA-F5	ACAACTAAGGAACAATGCTAAGGAAATCGG	1403
crRNA4-RT-RPA-R5	TTCCTCCCTATTCAACTTGGATTCTTCTGA	1518

**Table 3:** RT-qPCR and RT-RPA-CRISPR/CAS test results for clinical samples.

Method	RT-qPCR			RT-RPA-CRISPR/Cas		
Judgment	Total	Positive	Negative	Positive	Negative	Coincidence Rate
Positive samples	60	54	6	56	4	94.18%
Negative samples	26	4	22	7	19	

## Data Availability

All data generated and analyzed during this study are included in this published article.

## Supplementary Materials

The following supporting information can be downloaded at: https://www.mdpi.com/article/10.3390/v18010047/s1. Figure S1: Conserved analysis of the target across different subtypes of swine influenza viruses. Table S1: Determination of the critical value of the one-step test result; Table S2: Detection results of nucleic acids at different concentrations; Table S3: Results of RT-RPA-CRISPR/Cas12a and RT-PCR testing of clinical samples. Table S4: Sequence information of different subtypes of influenza viruses.
