# Supplementary material for: A Portable One-Tube Assay Integrating RT-RPA and CRISPR/Cas12a for Rapid Visual Detection of Eurasian Avian-like H1N1 Swine Influenza Virus in the Field
Source: Viruses. 2025 Dec 28;18(1):47. doi: 10.3390/v18010047 (PMC12846607; doi:10.3390/v18010047)
Supplement: Supplementary file 1 [file viruses-18-00047-s001.zip › viruses-4053961-supplementary.pdf]

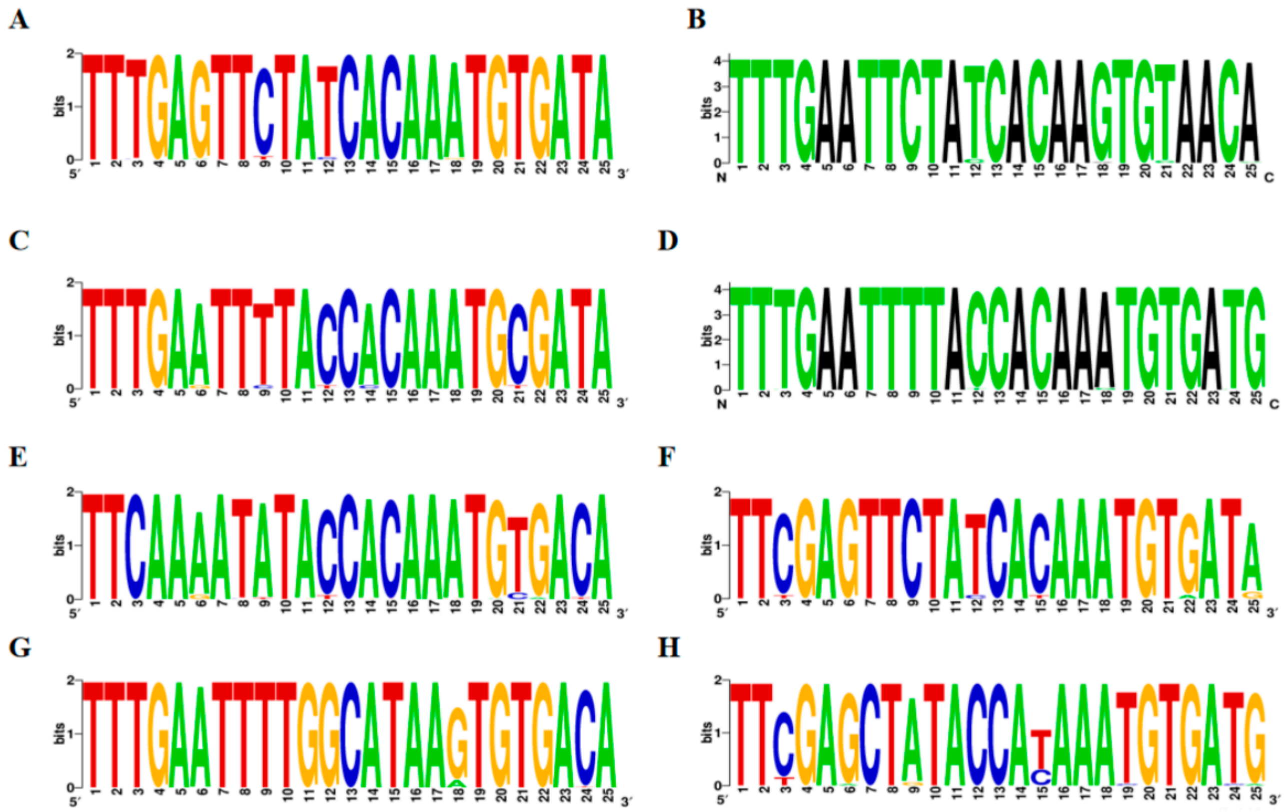

Figure S1. Homology alignment of the region targeted by crRNA4 on the HA gene

Panels A to G illustrate the conservation of the region targeted by crRNA4 on the hemagglutinin (HA) gene across different subtypes of swine influenza virus (SIV):A – Eurasian avian-like H1N1 SIV, B – Human-like H1N1 SIV, C – 2009 Pandemic H1N1 SIV, D – Classical H1N1 SIV, E – H3N2 SIV, F – H5N1 SIV, G – H6N6 SIV and H – H9N2 SIV.

Table S1. Determination of the critical value of the one-step test result

Detection results of 20 negative samples				Average	SD	critical value
10506.4	4169.8	1161.9	24378.1			
43405.1	1235.2	966.6	20356.5			
4879.6	2821.5	15560.1	30442.0	14863.8	12506.6	52383.45
29254.3	2594.4	972.5	11639.2			
21590.7	23646.0	22444.6	25251.2			

Table S2. Detection results of nucleic acids at different concentrations

Copies/μL	Fluorescence value/Repeat 1	Fluorescence value/Repeat 2	Fluorescence value/Repeat 3
2048	661929.75	690110.69	855358.25
1024	788575.13	729884.38	679159.56
512	614618.75	566144.25	728245.94
256	736556.69	772498.44	670908.63
128	495429.5	585449.63	591441.06
64	313686.25	505452.13	539472.63
32	539980.06	552256.69	561383.88
16	253649.55	485586.72	347603.34
8	395433.72	377688.78	381510.78
4	226370.14	74633.2	190065.83
2	246541.81	209612.08	598245.25
1	36263.25	437034.81	472229.09

Table S3. Results of RT-RPA-CRISPR/Cas12a and RT-PCR testing of clinical samples

Sample Number	VI/Judgment	CT/Judgment	RT-RPA-CRISPR/Cas12a/Judgment	Sample Number	VI/Judgment	CT/Judgment	RT-RPA-CRISPR/Cas12a/Judgment
1	N *	44.95/N	-880.87/N	21	N	-/N	-24726.29/N
2	N	-/N	-1033.20/N	22	N	-/N	64739.49/P
3	N	-/N	-1809.37/N	23	N	-/N	78568.86/P
4	N	-/N	-3407.92/N	24	N	-/N	-385.11/N
5	N	-/N	-1738.91/N	25	N	-/N	62475.01/P
6	N	21.09/P	423695.79/P	26	N	28.32/P	202633.01/P
7	N	-/N	-1242.06/N	27	P*	40.05/N	107166.64/P
8	N	-/N	-4515.13/N	28	P	42.92/N	62311.57/P
9	N	-/N	4766.65/N	29	P	-/N	50074.71/N
10	N	-/N	2595.36/N	30	P	35.62/P	313576.22/P
11	N	-/N	662.05/N	31	P	27.55/P	126240.02/P
12	N	-/N	-3387.77/N	32	P	23.07/P	720788.00/P
13	N	-/N	182.16/N	33	P	-/N	5205.33/N
14	N	25.24/P	232336.38/P	34	P	21.81/P	690738.44/P
15	N	-/N	11234.79/N	35	P	25.34/P	258494.02/P
16	N	-/N	9928.73/N	36	P	23.49/P	1037462.81/P
17	N	-/N	21584.26/N	37	P	32.46/P	238335.91/P
18	N	-/N	3784.77/N	38	P	22.58/P	339572.66/P
19	N	24.39/P	722637.75/P	39	P	25.30/P	178879.81/P
20	N	-/N	2192.90/N	40	P	40.53/N	-5843.70/N

Sample Number	VI/Judgment	CT/Judgment	RT-RPA-CRISPR/Cas12a/Judgment	Sample Number	VI/Judgment	CT/Judgment	RT-RPA-CRISPR/Cas12a/Judgment
41	P	26.83/P	315777.09/P	61	P	29.72/P	1063761.75/P
42	P	25.88/P	168542.53/P	62	P	24.72/P	884116.88/P
43	P	27.52/P	943143.88/P	63	P	28.16/P	249042.41/P
44	P	24.29/P	754496.31/P	64	P	28.69/P	143627.20/P
45	P	33.34/P	366774.13/P	65	P	27.17/P	202926.97/P
46	P	34.47/P	277430.38/P	66	P	35.95/P	106803.33/P
47	P	21.70/P	81717.09/P	67	P	33.84/P	840754.00/P
48	P	19.18/P	70602.53/P	68	P	32.42/P	777995.56/P
49	P	27.22/P	452166.00/P	69	P	35.62/P	395066.56/P
50	P	26.48/P	847256.06/P	70	P	31.72/P	746426.94/P
51	P	28.98/P	658762.88/P	71	P	29.92/P	177050.81/P
52	P	32.35/P	468841.06/P	72	P	37.31/P	1005987.44/P
53	P	32.16/P	105318.10/P	73	P	33.25/P	73805.35/P
54	P	30.79/P	115207.08/P	74	P	32.14/P	459808.25/P
55	P	31.04/P	155448.59/P	75	P	28.94/P	54292.20/P
56	P	20.86/P	853362.75/P	76	P	35.25/P	996968.00/P
57	P	22.01/P	301437.52/P	77	P	22.88/P	804548.06/P
58	P	33.27/P	150773.19/P	78	P	25.31/P	633322.50/P
59	P	34.40/P	730851.25/P	79	P	40.68/N	32170.19/N
60	P	34.87/P	781948.69/P	80	P	25.53/P	661566.00/P

Sample Number	VI/Judgment	CT/Judgment	RT-RPA-CRISPR/Cas12a/Judgment	Sample Number	VI/Judgment	CT/Judgment	RT-RPA-CRISPR/Cas12a/Judgment
81	P	29.66/P	552760.00/P	84	P	34.86/P	220237.61/P
82	P	24.51/P	734428.00/P	85	P	33.32/P	668502.75/P
83	P	28.15/P	176119.06/P	86	P	34.82/P	164974.45/P

*N represents a negative test result, and P represents a positive test result.

Table S4. Sequence information of different subtypes of influenza viruses.

Number	Type	HA Segment_ID	Name
1	Eurasian avian-like H1N1 SIV	EPI2048733	A/swine/Jilin/419/2015
2	Eurasian avian-like H1N1 SIV	EPI2049005	A/swine/Liaoning/995/2016
3	Eurasian avian-like H1N1 SIV	EPI2046853	A/swine/Guangxi/206/2016
4	Eurasian avian-like H1N1 SIV	EPI2049325	A/swine/Sichuan/952/2016
5	Eurasian avian-like H1N1 SIV	EPI2048405	A/swine/Hubei/2/2014
6	Eurasian avian-like H1N1 SIV	EPI2049173	A/swine/Sndong/540/2017
7	Eurasian avian-like H1N1 SIV	EPI2046757	A/swine/Guangxi/1164/2016
8	Eurasian avian-like H1N1 SIV	EPI2048925	A/swine/Liaoning/41/2018
9	Eurasian avian-like H1N1 SIV	EPI2049773	A/swine/Chongqing/171/2018
10	Eurasian avian-like H1N1 SIV	EPI2048149	A/swine/Hebei/309/2016
11	Eurasian avian-like H1N1 SIV	EPI2049549	A/swine/Tianjin/525/2018
12	Eurasian avian-like H1N1 SIV	EPI2049077	A/swine/Sanxi/330/2014
13	Eurasian avian-like H1N1 SIV	EPI2046689	A/swine/Guangdong/796/2019
14	Eurasian avian-like H1N1 SIV	EPI2049125	A/swine/Sndong/123/2018

15	Eurasian avian-like H1N1 SIV	EPI2048077	A/swine/Hebei/17/2016
16	Eurasian avian-like H1N1 SIV	EPI2048029	A/swine/Guizhou/477/2016
17	Eurasian avian-like H1N1 SIV	EPI2048837	A/swine/Liaoning/1100/2018
18	Eurasian avian-like H1N1 SIV	EPI2047869	A/swine/Guangxi/576/2018
19	Eurasian avian-like H1N1 SIV	EPI2048021	A/swine/Guizhou/440/2016
20	Eurasian avian-like H1N1 SIV	EPI2049533	A/swine/Tianjin/487/2018
21	Eurasian avian-like H1N1 SIV	EPI2048229	A/swine/Henan/189/2019
22	Eurasian avian-like H1N1 SIV	EPI2046845	A/swine/Guangxi/20/2015
23	Eurasian avian-like H1N1 SIV	EPI2048637	A/swine/Jiangxi/400/2015
24	Eurasian avian-like H1N1 SIV	EPI2049365	A/swine/Tianjin/13/2015
25	Eurasian avian-like H1N1 SIV	EPI2049157	A/swine/Sndong/414/2014
26	Eurasian avian-like H1N1 SIV	EPI2049357	A/swine/Tianjin/117/2019
27	Eurasian avian-like H1N1 SIV	EPI2048101	A/swine/Hebei/201/2016
28	Eurasian avian-like H1N1 SIV	EPI2049765	A/swine/Zhejiang/714/2019
29	Eurasian avian-like H1N1 SIV	EPI2046663	A/swine/Guangdong/642/2014
30	Eurasian avian-like H1N1 SIV	EPI2048557	A/swine/Jiangsu/562/2019
31	Eurasian avian-like H1N1 SIV	EPI2047981	A/swine/Guangxi/944/2016
32	Eurasian avian-like H1N1 SIV	EPI2048477	A/swine/Hunan/704/2017
33	Eurasian avian-like H1N1 SIV	EPI2048813	A/swine/Jilin/812/2017
34	Eurasian avian-like H1N1 SIV	EPI2049597	A/swine/Tianjin/66/2019
35	Eurasian avian-like H1N1 SIV	EPI2047845	A/swine/Guangxi/439/2018
36	Eurasian avian-like H1N1 SIV	EPI2049421	A/swine/Tianjin/212/2016
37	Eurasian avian-like H1N1 SIV	EPI2049021	A/swine/Sanxi/1199/2016

38	Eurasian avian-like H1N1 SIV	EPI2048845	A/swine/Liaoning/1152/2018
39	Eurasian avian-like H1N1 SIV	EPI2049445	A/swine/Tianjin/282/2018
40	Eurasian avian-like H1N1 SIV	EPI2049517	A/swine/Tianjin/448/2019
41	Eurasian avian-like H1N1 SIV	EPI2049309	A/swine/Sichuan/643/2017
42	Eurasian avian-like H1N1 SIV	EPI2048085	A/swine/Hebei/177/2016
43	Eurasian avian-like H1N1 SIV	EPI554315	A/swine/Guangdong/6/2013
44	Eurasian avian-like H1N1 SIV	EPI2049029	A/swine/Sanxi/1254/2016
45	Eurasian avian-like H1N1 SIV	EPI2048285	A/swine/Henan/259/2019
46	Eurasian avian-like H1N1 SIV	EPI2049333	A/swine/Sichuan/968/2016
47	Eurasian avian-like H1N1 SIV	EPI2046469	A/swine/Chongqing/104/2016
48	Eurasian avian-like H1N1 SIV	EPI2049493	A/swine/Tianjin/380/2018
49	Eurasian avian-like H1N1 SIV	EPI2049069	A/swine/Sanxi/1644/2016
50	Eurasian avian-like H1N1 SIV	EPI2049677	A/swine/Tianjin/787/2019
51	Eurasian avian-like H1N1 SIV	EPI2047917	A/swine/Guangxi/717/2019
52	Eurasian avian-like H1N1 SIV	EPI2049469	A/swine/Tianjin/312/2016
53	Eurasian avian-like H1N1 SIV	EPI554395	A/swine/Hunan/153/2013
54	Eurasian avian-like H1N1 SIV	EPI2049717	A/swine/Zhejiang/132/2019
55	Eurasian avian-like H1N1 SIV	EPI554523	A/swine/Tianjin/45/2011
56	Eurasian avian-like H1N1 SIV	EPI2047813	A/swine/Guangxi/370/2016
57	Eurasian avian-like H1N1 SIV	EPI2047997	A/swine/Guangxi/960/2016
58	Eurasian avian-like H1N1 SIV	EPI2048613	A/swine/Jiangxi/285/2016
59	Eurasian avian-like H1N1 SIV	EPI2048429	A/swine/Hubei/618/2019
60	Eurasian avian-like H1N1 SIV	EPI2049581	A/swine/Tianjin/632/2019

61	Eurasian avian-like H1N1 SIV	EPI2048853	A/swine/Liaoning/18/2018
62	Eurasian avian-like H1N1 SIV	EPI2049373	A/swine/Tianjin/138/2016
63	Eurasian avian-like H1N1 SIV	EPI2046861	A/swine/Guangxi/233/2018
64	Eurasian avian-like H1N1 SIV	EPI2048941	A/swine/Liaoning/438/2019
65	Eurasian avian-like H1N1 SIV	EPI2049397	A/swine/Tianjin/171/2019
66	Eurasian avian-like H1N1 SIV	EPI2048117	A/swine/Hebei/231/2016
67	Eurasian avian-like H1N1 SIV	EPI2048965	A/swine/Liaoning/721/2019
68	Eurasian avian-like H1N1 SIV	EPI2046837	A/swine/Guangxi/166/2016
69	Eurasian avian-like H1N1 SIV	EPI2049277	A/swine/Sichuan/189/2014
70	Eurasian avian-like H1N1 SIV	EPI2046518	A/swine/Chongqing/426/2016
71	Eurasian avian-like H1N1 SIV	EPI2048005	A/swine/Guizhou/132/2016
72	Eurasian avian-like H1N1 SIV	EPI2049565	A/swine/Tianjin/575/2019
73	Eurasian avian-like H1N1 SIV	EPI2049637	A/swine/Tianjin/741/2018
74	Eurasian avian-like H1N1 SIV	EPI2048885	A/swine/Liaoning/248/2018
75	Eurasian avian-like H1N1 SIV	EPI2046526	A/swine/Chongqing/444/2017
76	Eurasian avian-like H1N1 SIV	EPI2048781	A/swine/Jilin/639/2017
77	Eurasian avian-like H1N1 SIV	EPI2048933	A/swine/Liaoning/423/2019
78	Eurasian avian-like H1N1 SIV	EPI2048141	A/swine/Hebei/294/2016
79	Eurasian avian-like H1N1 SIV	EPI554347	A/swine/Guangxi/332/2011
80	Eurasian avian-like H1N1 SIV	EPI2049165	A/swine/Sndong/451/2017
81	Eurasian avian-like H1N1 SIV	EPI2049485	A/swine/Tianjin/332/2014
82	Eurasian avian-like H1N1 SIV	EPI2046671	A/swine/Guangdong/702/2014
83	Eurasian avian-like H1N1 SIV	EPI2048693	A/swine/Jilin/280/2017

84	Eurasian avian-like H1N1 SIV	EPI2046501	A/swine/Chongqing/241/2018
85	Eurasian avian-like H1N1 SIV	EPI2049381	A/swine/Tianjin/144/2014
86	Eurasian avian-like H1N1 SIV	EPI2049085	A/swine/Sanxi/428/2017
87	Eurasian avian-like H1N1 SIV	EPI2049573	A/swine/Tianjin/624/2018
88	Eurasian avian-like H1N1 SIV	EPI554371	A/swine/Heilongjiang/30/2012
89	Eurasian avian-like H1N1 SIV	EPI2049109	A/swine/Sanxi/726/2017
90	Eurasian avian-like H1N1 SIV	EPI2048293	A/swine/Henan/268/2019
91	Eurasian avian-like H1N1 SIV	EPI2048589	A/swine/Jiangsu/702/2019
92	Eurasian avian-like H1N1 SIV	EPI2048253	A/swine/Henan/207/2019
93	Eurasian avian-like H1N1 SIV	EPI2048157	A/swine/Hebei/317/2016
94	Eurasian avian-like H1N1 SIV	EPI2049797	A/swine/Guangxi/755/2017
95	Eurasian avian-like H1N1 SIV	EPI2048805	A/swine/Jilin/809/2017
96	Eurasian avian-like H1N1 SIV	EPI554291	A/swine/Guangdong/30/2013
97	Eurasian avian-like H1N1 SIV	EPI2048917	A/swine/Liaoning/357/2018
98	Eurasian avian-like H1N1 SIV	EPI2049429	A/swine/Tianjin/217/2014
99	Eurasian avian-like H1N1 SIV	EPI2049189	A/swine/Sndong/60/2019
100	Eurasian avian-like H1N1 SIV	EPI1987749	A/swine/Guizhou/828/2016
101	Eurasian avian-like H1N1 SIV	EPI554307	A/swine/Guangdong/5/2013
102	Eurasian avian-like H1N1 SIV	EPI554379	A/swine/Heilongjiang/B106/2011
103	Eurasian avian-like H1N1 SIV	EPI2049629	A/swine/Tianjin/708/2019
104	Eurasian avian-like H1N1 SIV	EPI2049709	A/swine/Tianjin/947/2016
105	Eurasian avian-like H1N1 SIV	EPI2048749	A/swine/Jilin/489/2015
106	Eurasian avian-like H1N1 SIV	EPI2048221	A/swine/Henan/189/2014

107	Eurasian avian-like H1N1 SIV	EPI2049461	A/swine/Tianjin/301/2018
108	Eurasian avian-like H1N1 SIV	EPI2049685	A/swine/Tianjin/800/2018
109	Eurasian avian-like H1N1 SIV	EPI2047853	A/swine/Guangxi/56/2016
110	Eurasian avian-like H1N1 SIV	EPI2048669	A/swine/Jiangxi/699/2017
111	Eurasian avian-like H1N1 SIV	EPI2049205	A/swine/Sndong/633/2019
112	Eurasian avian-like H1N1 SIV	EPI2049557	A/swine/Tianjin/536/2019
113	Eurasian avian-like H1N1 SIV	EPI2049501	A/swine/Tianjin/396/2016
114	Eurasian avian-like H1N1 SIV	EPI2046885	A/swine/Guangxi/276/2014
115	Eurasian avian-like H1N1 SIV	EPI2047893	A/swine/Guangxi/669/2016
116	Eurasian avian-like H1N1 SIV	EPI2048133	A/swine/Hebei/292/2018
117	Eurasian avian-like H1N1 SIV	EPI2048549	A/swine/Jiangsu/547/2017
118	Eurasian avian-like H1N1 SIV	EPI2049653	A/swine/Tianjin/781/2019
119	Eurasian avian-like H1N1 SIV	EPI2047925	A/swine/Guangxi/749/2017
120	Eurasian avian-like H1N1 SIV	EPI2046485	A/swine/Chongqing/184/2016
121	Eurasian avian-like H1N1 SIV	EPI2048349	A/swine/Henan/656/2019
122	Eurasian avian-like H1N1 SIV	EPI2049149	A/swine/Sndong/363/2017
123	Eurasian avian-like H1N1 SIV	EPI2048869	A/swine/Liaoning/225/2017
124	Eurasian avian-like H1N1 SIV	EPI2048125	A/swine/Hebei/250/2016
125	Eurasian avian-like H1N1 SIV	EPI2048093	A/swine/Hebei/197/2016
126	Eurasian avian-like H1N1 SIV	EPI2048701	A/swine/Jilin/289/2019
127	Eurasian avian-like H1N1 SIV	EPI2049181	A/swine/Sndong/573/2014
128	Eurasian avian-like H1N1 SIV	EPI2046577	A/swine/Chongqing/709/2017
129	Eurasian avian-like H1N1 SIV	EPI2049541	A/swine/Tianjin/490/2018

130	Eurasian avian-like H1N1 SIV	EPI2048685	A/swine/Jilin/217/2017
131	Eurasian avian-like H1N1 SIV	EPI554515	A/swine/Tianjin/42/2011
132	Eurasian avian-like H1N1 SIV	EPI2049261	A/swine/Sichuan/1142/2017
133	Eurasian avian-like H1N1 SIV	EPI2048645	A/swine/Jiangxi/471/2016
134	Eurasian avian-like H1N1 SIV	EPI2046603	A/swine/Guangdong/132/2019
135	Eurasian avian-like H1N1 SIV	EPI2049821	A/swine/Henan/600/2016
136	Eurasian avian-like H1N1 SIV	EPI2047789	A/swine/Guangxi/316/2016
137	Eurasian avian-like H1N1 SIV	EPI2046732	A/swine/Guangxi/1080/2013
138	Eurasian avian-like H1N1 SIV	EPI2049477	A/swine/Tianjin/322/2018
139	Eurasian avian-like H1N1 SIV	EPI2048757	A/swine/Jilin/515/2015
140	Eurasian avian-like H1N1 SIV	EPI2048277	A/swine/Henan/231/2019
141	Eurasian avian-like H1N1 SIV	EPI2048517	A/swine/Jiangsu/45/2019
142	Eurasian avian-like H1N1 SIV	EPI2048653	A/swine/Jiangxi/479/2017
143	Eurasian avian-like H1N1 SIV	EPI2047909	A/swine/Guangxi/695/2016
144	Eurasian avian-like H1N1 SIV	EPI2046781	A/swine/Guangxi/1183/2016
145	Eurasian avian-like H1N1 SIV	EPI2048045	A/swine/Hebei/101/2016
146	Eurasian avian-like H1N1 SIV	EPI2049285	A/swine/Sichuan/401/2017
147	Eurasian avian-like H1N1 SIV	EPI2049613	A/swine/Tianjin/675/2019
148	Eurasian avian-like H1N1 SIV	EPI2049733	A/swine/Zhejiang/33/2019
149	Eurasian avian-like H1N1 SIV	EPI2046392	A/swine/Anhui/156/2013
150	Eurasian avian-like H1N1 SIV	EPI2046400	A/swine/Anhui/335/2018
151	Eurasian avian-like H1N1 SIV	EPI2049213	A/swine/Sndong/75/2018
152	Eurasian avian-like H1N1 SIV	EPI2048373	A/swine/Henan/771/2018

153	Eurasian avian-like H1N1 SIV	EPI2048389	A/swine/Henan/804/2019
154	Eurasian avian-like H1N1 SIV	EPI2048469	A/swine/Hunan/580/2017
155	Eurasian avian-like H1N1 SIV	EPI2049061	A/swine/Snnxi/1454/2016
156	Eurasian avian-like H1N1 SIV	EPI2047829	A/swine/Guangxi/404/2018
157	Eurasian avian-like H1N1 SIV	EPI2049269	A/swine/Sichuan/165/2017
158	Eurasian avian-like H1N1 SIV	EPI2048821	A/swine/Liaoning/10/2015
159	Eurasian avian-like H1N1 SIV	EPI2047949	A/swine/Guangxi/833/2016
160	Eurasian avian-like H1N1 SIV	EPI2048325	A/swine/Henan/57/2019
161	Eurasian avian-like H1N1 SIV	EPI2046655	A/swine/Guangdong/634/2014
162	Eurasian avian-like H1N1 SIV	EPI2048053	A/swine/Hebei/11/2016
163	Eurasian avian-like H1N1 SIV	EPI2049813	A/swine/Henan/599/2016
164	Eurasian avian-like H1N1 SIV	EPI2048173	A/swine/Hebei/60/2018
165	Eurasian avian-like H1N1 SIV	EPI554403	A/swine/Hunan/196/2011
166	Eurasian avian-like H1N1 SIV	EPI2048677	A/swine/Jilin/164/2019
167	Eurasian avian-like H1N1 SIV	EPI2048533	A/swine/Jiangsu/486/2019
168	Eurasian avian-like H1N1 SIV	EPI2048629	A/swine/Jiangxi/330/2016
169	Eurasian avian-like H1N1 SIV	EPI2047885	A/swine/Guangxi/650/2018
170	Eurasian avian-like H1N1 SIV	EPI2049725	A/swine/Zhejiang/1836/2016
171	Eurasian avian-like H1N1 SIV	EPI2046797	A/swine/Guangxi/1253/2016
172	Eurasian avian-like H1N1 SIV	EPI2048237	A/swine/Henan/204/2019
173	Eurasian avian-like H1N1 SIV	EPI2049589	A/swine/Tianjin/645/2019
174	Eurasian avian-like H1N1 SIV	EPI2048109	A/swine/Hebei/23/2016
175	Eurasian avian-like H1N1 SIV	EPI2049101	A/swine/Sanxi/464/2017

176	Eurasian avian-like H1N1 SIV	EPI2046821	A/swine/Guangxi/1392/2016
177	Eurasian avian-like H1N1 SIV	EPI2048877	A/swine/Liaoning/23/2015
178	Eurasian avian-like H1N1 SIV	EPI2049749	A/swine/Zhejiang/379/2019
179	Eurasian avian-like H1N1 SIV	EPI2048797	A/swine/Jilin/723/2017
180	Eurasian avian-like H1N1 SIV	EPI2047877	A/swine/Guangxi/599/2016
181	Eurasian avian-like H1N1 SIV	EPI2048301	A/swine/Henan/379/2019
182	Eurasian avian-like H1N1 SIV	EPI2049837	A/swine/Yunnan/221/2018
183	Eurasian avian-like H1N1 SIV	EPI2046646	A/swine/Guangdong/531/2014
184	Eurasian avian-like H1N1 SIV	EPI2047941	A/swine/Guangxi/78/2016
185	Eurasian avian-like H1N1 SIV	EPI2049437	A/swine/Tianjin/238/2015
186	Eurasian avian-like H1N1 SIV	EPI2047821	A/swine/Guangxi/391/2013
187	Eurasian avian-like H1N1 SIV	EPI2049317	A/swine/Sichuan/9/2017
188	Eurasian avian-like H1N1 SIV	EPI2049669	A/swine/Tianjin/785/2019
189	Eurasian avian-like H1N1 SIV	EPI2046723	A/swine/Guangxi/1047/2016
190	Eurasian avian-like H1N1 SIV	EPI2048773	A/swine/Jilin/603/2015
191	Eurasian avian-like H1N1 SIV	EPI2048989	A/swine/Liaoning/972/2016
192	Eurasian avian-like H1N1 SIV	EPI2048013	A/swine/Guizhou/224/2014
193	Eurasian avian-like H1N1 SIV	EPI2048893	A/swine/Liaoning/267/2017
194	Eurasian avian-like H1N1 SIV	EPI2048461	A/swine/Hunan/53/2017
195	Eurasian avian-like H1N1 SIV	EPI2049661	A/swine/Tianjin/784/2018
196	Eurasian avian-like H1N1 SIV	EPI2046877	A/swine/Guangxi/264/2019
197	Eurasian avian-like H1N1 SIV	EPI2049197	A/swine/Sndong/612/2017
198	Eurasian avian-like H1N1 SIV	EPI2049829	A/swine/Jiangxi/186/2017

199	Eurasian avian-like H1N1 SIV	EPI2046621	A/swine/Guangdong/436/2014
200	Eurasian avian-like H1N1 SIV	EPI2049701	A/swine/Tianjin/848/2016
201	Eurasian avian-like H1N1 SIV	EPI2048765	A/swine/Jilin/588/2019
202	Eurasian avian-like H1N1 SIV	EPI2049013	A/swine/Sanxi/1150/2016
203	Eurasian avian-like H1N1 SIV	EPI2048445	A/swine/Hunan/319/2017
204	Eurasian avian-like H1N1 SIV	EPI2048741	A/swine/Jilin/45/2019
205	Eurasian avian-like H1N1 SIV	EPI2048069	A/swine/Hebei/168/2016
206	Eurasian avian-like H1N1 SIV	EPI2048365	A/swine/Henan/764/2019
207	Eurasian avian-like H1N1 SIV	EPI2048261	A/swine/Henan/208/2019
208	Eurasian avian-like H1N1 SIV	EPI2046595	A/swine/Chongqing/91/2018
209	Eurasian avian-like H1N1 SIV	EPI2049605	A/swine/Tianjin/660/2019
210	Eurasian avian-like H1N1 SIV	EPI2048341	A/swine/Henan/606/2018
211	Eurasian avian-like H1N1 SIV	EPI2048957	A/swine/Liaoning/53/2017
212	Eurasian avian-like H1N1 SIV	EPI2047861	A/swine/Guangxi/575/2016
213	Eurasian avian-like H1N1 SIV	EPI554459	A/swine/Jiangsu/49/2012
214	Eurasian avian-like H1N1 SIV	EPI2046637	A/swine/Guangdong/473/2014
215	Eurasian avian-like H1N1 SIV	EPI2046510	A/swine/Chongqing/244/2016
216	Eurasian avian-like H1N1 SIV	EPI2048789	A/swine/Jilin/683/2019
217	Eurasian avian-like H1N1 SIV	EPI2049221	A/swine/Sndong/807/2014
218	Eurasian avian-like H1N1 SIV	EPI2048317	A/swine/Henan/540/2019
219	Eurasian avian-like H1N1 SIV	EPI2049389	A/swine/Tianjin/159/2016
220	Eurasian avian-like H1N1 SIV	EPI2046697	A/swine/Guangxi/10/2016
221	Eurasian avian-like H1N1 SIV	EPI2049405	A/swine/Tianjin/18/2018

222	Eurasian avian-like H1N1 SIV	EPI2049253	A/swine/Sichuan/1134/2016
223	Eurasian avian-like H1N1 SIV	EPI2047765	A/swine/Guangxi/276/2018
224	Eurasian avian-like H1N1 SIV	EPI2046461	A/swine/Anhui/597/2015
225	Eurasian avian-like H1N1 SIV	EPI2048213	A/swine/Henan/186/2014
226	Eurasian avian-like H1N1 SIV	EPI2048909	A/swine/Liaoning/337/2019
227	Eurasian avian-like H1N1 SIV	EPI2046561	A/swine/Chongqing/705/2016
228	Eurasian avian-like H1N1 SIV	EPI2047901	A/swine/Guangxi/683/2013
229	Eurasian avian-like H1N1 SIV	EPI554331	A/swine/Guangxi/18/2011
230	Eurasian avian-like H1N1 SIV	EPI2046829	A/swine/Guangxi/148/2014
231	Eurasian avian-like H1N1 SIV	EPI2048949	A/swine/Liaoning/445/2018
232	Eurasian avian-like H1N1 SIV	EPI554419	A/swine/Hunan/30/2013
233	Eurasian avian-like H1N1 SIV	EPI554339	A/swine/Guangxi/24/2011
234	Eurasian avian-like H1N1 SIV	EPI2047957	A/swine/Guangxi/885/2016
235	Eurasian avian-like H1N1 SIV	EPI2049117	A/swine/Sndong/10/2017
236	Eurasian avian-like H1N1 SIV	EPI2048573	A/swine/Jiangsu/648/2019
237	Eurasian avian-like H1N1 SIV	EPI2046706	A/swine/Guangxi/1017/2016
238	Eurasian avian-like H1N1 SIV	EPI2047973	A/swine/Guangxi/927/2016
239	Eurasian avian-like H1N1 SIV	EPI2046765	A/swine/Guangxi/1169/2016
240	Eurasian avian-like H1N1 SIV	EPI554475	A/swine/Liaoning/225/2012
241	Eurasian avian-like H1N1 SIV	EPI2048541	A/swine/Jiangsu/537/2019
242	Eurasian avian-like H1N1 SIV	EPI2048181	A/swine/Hebei/726/2017
243	Eurasian avian-like H1N1 SIV	EPI2047965	A/swine/Guangxi/926/2016
244	Eurasian avian-like H1N1 SIV	EPI2048189	A/swine/Heilongjiang/1085/2015

245	Eurasian avian-like H1N1 SIV	EPI554387	A/swine/Henan/232/2011
246	Eurasian avian-like H1N1 SIV	EPI2046445	A/swine/Anhui/583/2017
247	Eurasian avian-like H1N1 SIV	EPI2047805	A/swine/Guangxi/360/2016
248	Eurasian avian-like H1N1 SIV	EPI2046773	A/swine/Guangxi/118/2017
249	Eurasian avian-like H1N1 SIV	EPI2049413	A/swine/Tianjin/182/2019
250	Eurasian avian-like H1N1 SIV	EPI2049293	A/swine/Sichuan/479/2017
251	Eurasian avian-like H1N1 SIV	EPI554355	A/swine/Hebei/148/2012
252	Eurasian avian-like H1N1 SIV	EPI2046551	A/swine/Chongqing/622/2016
253	Eurasian avian-like H1N1 SIV	EPI2048981	A/swine/Liaoning/940/2018
254	Eurasian avian-like H1N1 SIV	EPI2046408	A/swine/Anhui/53/2018
255	Eurasian avian-like H1N1 SIV	EPI2048861	A/swine/Liaoning/217/2019
256	Eurasian avian-like H1N1 SIV	EPI2048493	A/swine/Jiangsu/103/2015
257	Eurasian avian-like H1N1 SIV	EPI2048829	A/swine/Liaoning/1011/2016
258	Eurasian avian-like H1N1 SIV	EPI2048309	A/swine/Henan/465/2019
259	Eurasian avian-like H1N1 SIV	EPI554275	A/swine/Guangdong/109/2013
260	Eurasian avian-like H1N1 SIV	EPI2046748	A/swine/Guangxi/1119/2016
261	Eurasian avian-like H1N1 SIV	EPI2049621	A/swine/Tianjin/696/2019
262	Eurasian avian-like H1N1 SIV	EPI2049509	A/swine/Tianjin/42/2019
263	Eurasian avian-like H1N1 SIV	EPI554283	A/swine/Guangdong/2/2013
264	Eurasian avian-like H1N1 SIV	EPI2048197	A/swine/Heilongjiang/1092/2015
265	Eurasian avian-like H1N1 SIV	EPI2046681	A/swine/Guangdong/759/2019
266	Eurasian avian-like H1N1 SIV	EPI2047989	A/swine/Guangxi/957/2016
267	Eurasian avian-like H1N1 SIV	EPI2046813	A/swine/Guangxi/1360/2016

268	Eurasian avian-like H1N1 SIV	EPI2049229	A/swine/Sichuan/1026/2016
269	Eurasian avian-like H1N1 SIV	EPI2048421	A/swine/Hubei/580/2019
270	Eurasian avian-like H1N1 SIV	EPI554483	A/swine/Liaoning/96/2012
271	Eurasian avian-like H1N1 SIV	EPI554451	A/swine/Jiangsu/38/2011
272	Eurasian avian-like H1N1 SIV	EPI554363	A/swine/Heilongjiang/27/2012
273	Eurasian avian-like H1N1 SIV	EPI2047933	A/swine/Guangxi/777/2016
274	Eurasian avian-like H1N1 SIV	EPI2048437	A/swine/Hunan/269/2016
275	Eurasian avian-like H1N1 SIV	EPI2048709	A/swine/Jilin/318/2017
276	Eurasian avian-like H1N1 SIV	EPI554531	A/swine/Tianjin/47/2011
277	Eurasian avian-like H1N1 SIV	EPI2049053	A/swine/Sanxi/1392/2016
278	Eurasian avian-like H1N1 SIV	EPI2048413	A/swine/Hubei/359/2014
279	Eurasian avian-like H1N1 SIV	EPI2048205	A/swine/Heilongjiang/1130/2015
280	Eurasian avian-like H1N1 SIV	EPI554299	A/swine/Guangdong/32/2013
281	Eurasian avian-like H1N1 SIV	EPI554467	A/swine/Liaoning/117/2012
282	Eurasian avian-like H1N1 SIV	EPI2046740	A/swine/Guangxi/1089/2016
283	Eurasian avian-like H1N1 SIV	EPI2048357	A/swine/Guangxi/1170/2013
284	Eurasian avian-like H1N1 SIV	EPI2048037	A/swine/Guizhou/699/2016
285	Eurasian avian-like H1N1 SIV	EPI2048397	A/swine/Hubei/183/2014
286	Eurasian avian-like H1N1 SIV	EPI2046535	A/swine/Chongqing/445/2018
287	Eurasian avian-like H1N1 SIV	EPI2048453	A/swine/Hunan/519/2017
288	Eurasian avian-like H1N1 SIV	EPI2049093	A/swine/Sanxi/44/2017
289	Eurasian avian-like H1N1 SIV	EPI554411	A/swine/Hunan/285/2013
290	Eurasian avian-like H1N1 SIV	EPI2046493	A/swine/Chongqing/241/2017

291	Eurasian avian-like H1N1 SIV	EPI2049789	A/swine/Chongqing/581/2018
292	Eurasian avian-like H1N1 SIV	EPI2047797	A/swine/Guangxi/324/2016
293	Eurasian avian-like H1N1 SIV	EPI2048565	A/swine/Jiangsu/629/2019
294	Eurasian avian-like H1N1 SIV	EPI2046569	A/swine/Chongqing/708/2017
295	Eurasian avian-like H1N1 SIV	EPI2046715	A/swine/Guangxi/1041/2016
296	Eurasian avian-like H1N1 SIV	EPI2048525	A/swine/Jiangsu/462/2017
297	Eurasian avian-like H1N1 SIV	EPI2049037	A/swine/Sanxi/1309/2016
298	Eurasian avian-like H1N1 SIV	EPI2049805	A/swine/Henan/483/2016
299	Eurasian avian-like H1N1 SIV	EPI2046629	A/swine/Guangdong/457/2014
300	Eurasian avian-like H1N1 SIV	EPI2049741	A/swine/Zhejiang/334/2016
301	Eurasian avian-like H1N1 SIV	EPI2049453	A/swine/Tianjin/294/2018
302	Eurasian avian-like H1N1 SIV	EPI554435	A/swine/Hunan/482/2013
303	Eurasian avian-like H1N1 SIV	EPI554443	A/swine/Hunan/951/2013
304	Eurasian avian-like H1N1 SIV	EPI2046586	A/swine/Chongqing/88/2017
305	Eurasian avian-like H1N1 SIV	EPI2048333	A/swine/Henan/579/2019
306	Eurasian avian-like H1N1 SIV	EPI2049237	A/swine/Sichuan/1055/2016
307	Eurasian avian-like H1N1 SIV	EPI2048725	A/swine/Jilin/377/2019
308	Eurasian avian-like H1N1 SIV	EPI2048605	A/swine/Jiangxi/261/2016
309	Eurasian avian-like H1N1 SIV	EPI554491	A/swine/Sichuan/172/2012
310	Eurasian avian-like H1N1 SIV	EPI2048381	A/swine/Henan/776/2019
311	Eurasian avian-like H1N1 SIV	EPI2047773	A/swine/Guangxi/293/2016
312	Eurasian avian-like H1N1 SIV	EPI2049525	A/swine/Tianjin/483/2019
313	Eurasian avian-like H1N1 SIV	EPI2047837	A/swine/Guangxi/416/2016

314	Eurasian avian-like H1N1 SIV	EPI2049693	A/swine/Tianjin/821/2016
315	Eurasian avian-like H1N1 SIV	EPI2048597	A/swine/Jiangsu/735/2019
316	Eurasian avian-like H1N1 SIV	EPI2046869	A/swine/Guangxi/264/2014
317	Eurasian avian-like H1N1 SIV	EPI2048581	A/swine/Jiangsu/674/2019
318	Eurasian avian-like H1N1 SIV	EPI554323	A/swine/Guangdong/95/2013
319	Eurasian avian-like H1N1 SIV	EPI2049301	A/swine/Sichuan/492/2017
320	2009 Pandemic H1N1 SIV	EPI121186	A/swine/Shandong/731/2009
321	2009 Pandemic H1N1 SIV	EPI89637	A/swine/Nanchang/3/2010
322	2009 Pandemic H1N1 SIV	EPI104327	A/swine/Jangsu/295/2010
323	2009 Pandemic H1N1 SIV	EPI89639	A/swine/Nanchang/5/2010
324	2009 Pandemic H1N1 SIV	EPI89638	A/swine/Nanchang/F9/2010
325	2009 Pandemic H1N1 SIV	EPI104329	A/swine/Jangsu/504/2010
326	2009 Pandemic H1N1 SIV	EPI89641	A/swine/Shandong/N1/2009
327	2009 Pandemic H1N1 SIV	EPI12957484	A/swine/Guangxi/717/2019
328	2009 Pandemic H1N1 SIV	EPI256362	A/swine/Shandong/POS3730/2016
329	2009 Pandemic H1N1 SIV	EPI104328	A/swine/Jangsu/434/2010
330	2009 Pandemic H1N1 SIV	EPI89640	A/swine/Nanchang/6/2010
331	2009 Pandemic H1N1 SIV	EPI256361	A/swine/Shandong/POS3718/2016
332	2009 Pandemic H1N1 SIV	EPI104299	A/swine/Jangsu/46/2010
333	2009 Pandemic H1N1 SIV	EPI256360	A/swine/Shandong/POS3717/2016
334	2009 Pandemic H1N1 SIV	EPI104301	A/swine/Jangsu/49/2010
335	2009 Pandemic H1N1 SIV	EPI104300	A/swine/Jangsu/48/2010
336	2009 Pandemic H1N1 SIV	EPI104302	A/swine/Jangsu/285/2010

337	2009 Pandemic H1N1 SIV	EPI104242	A/swine/Jangsu/38/2010
338	2009 Pandemic H1N1 SIV	EPI103830	A/swine/Heilongjiang/44/2009
339	2009 Pandemic H1N1 SIV	EPI103833	A/swine/Heilongjiang/105/2009
340	2009 Pandemic H1N1 SIV	EPI103835	A/swine/Yunnan/74/2009
341	Classical H1N1 SIV	HM210852.1	A/swine/Guangdong/09/2009(H1N1)
342	Classical H1N1 SIV	HM223594.1	A/swine/Guangdong/02/2008(H1N1)
343	Classical H1N1 SIV	HM135403.1	A/swine/Guangdong/11/2009(H1N1)
344	Classical H1N1 SIV	HQ880614.1	A/swine/Guangdong/L6/2009(H1N1)
345	Classical H1N1 SIV	DQ058215.1	A/swine/Guangdong/2/01(H1N1)
346	Classical H1N1 SIV	HM215151.1	A/swine/Guangdong/06/2009(H1N1)
347	Classical H1N1 SIV	HM223586.1	A/swine/Guangdong/07/2008(H1N1)
348	Classical H1N1 SIV	GQ422385.1	A/swine/Guangdong/103/2002(H1N1)
349	Classical H1N1 SIV	EU502885.1	A/swine/Shanghai/2/2005(H1N1)
350	Classical H1N1 SIV	CY089867.1	A/swine/Guangdong/34/2006(H1N1)
351	Classical H1N1 SIV	GU086033.1	A/swine/Guangdong/446/2006(H1N1)
352	Classical H1N1 SIV	GU086041.1	A/swine/Guangdong/611/2006(H1N1)
353	Classical H1N1 SIV	FJ789832.1	A/swine/Shanghai/3/2005(H1N1)
354	Classical H1N1 SIV	GU086017.1	A/swine/Guangdong/109/2006(H1N1)
355	Classical H1N1 SIV	GU086049.1	A/swine/Guangdong/628/2006(H1N1)
356	Classical H1N1 SIV	GU086025.1	A/swine/Guangdong/322/2006(H1N1)
357	Classical H1N1 SIV	GU646023.1	A/swine/Shandong/1112/2008(H1N1)
358	Classical H1N1 SIV	GU646030.1	A/swine/Shandong/1123/2008(H1N1)
359	Classical H1N1 SIV	EU502884.1	A/swine/Shanghai/1/2005(H1N1)

360	Classical H1N1 SIV	GQ452272.1	A/swine/Guangdong/2/2009(H1N1)
361	Classical H1N1 SIV	HQ541656.1	A/swine/Guangxi/12/2005(H1N1)
362	Classical H1N1 SIV	MT410579.1	A/swine/Guangdong/1/2011(H1N1)
363	Classical H1N1 SIV	KP186011.1	A/Swine/Guangdong/2004(H1N1)
364	Classical H1N1 SIV	FJ536842.1	A/swine/Guangdong/1/2005(H1N1)
365	Classical H1N1 SIV	AY852271.1	A/swine/Guangdong/711/2001(H1N1)
366	Classical H1N1 SIV	CY087112.1	A/swine/Hong Kong/2592/1994(H1N1)
367	Classical H1N1 SIV	CY087120.1	A/swine/Hong Kong/2603/1994(H1N1)
368	Classical H1N1 SIV	CY087096.1	A/swine/Hong Kong/2507/1994(H1N1)
369	Classical H1N1 SIV	CY087056.1	A/swine/Hong Kong/2422/1994(H1N1)
370	Classical H1N1 SIV	CY087072.1	A/swine/Hong Kong/2445/1994(H1N1)
371	Classical H1N1 SIV	CY087064.1	A/swine/Hong Kong/2428/1994(H1N1)
372	Classical H1N1 SIV	CY087080.1	A/swine/Hong Kong/2461/1994(H1N1)
373	Classical H1N1 SIV	CY085121.1	A/swine/Hong Kong/1937/1994(H1N1)
374	Classical H1N1 SIV	CY087048.1	A/swine/Hong Kong/2161/1994(H1N1)
375	Classical H1N1 SIV	CY087088.1	A/swine/Hong Kong/2503/1994(H1N1)
376	Classical H1N1 SIV	U45452.1	A/swine/Hong Kong/273/1994(H1N1)
377	Classical H1N1 SIV	KF057118.1	A/swine/Beijing/216/1992(H1N1)
378	Classical H1N1 SIV	KF057117.1	A/swine/Beijing/156/1991(H1N1)
379	Classical H1N1 SIV	CY035070.1	A/swine/Memphis/1/1990(H1N1)
380	Classical H1N1 SIV	CY027155.1	A/swine/Iowa/24297/1991(H1N1)
381	Classical H1N1 SIV	CY022477.1	A/swine/Maryland/23239/1991(H1N1)
382	Classical H1N1 SIV	CY028780.1	A/swine/California/T9001707/1991(H1N1)

383	Classical H1N1 SIV	CY084961.1	A/swine/Hong Kong/128/1993(H1N1)
384	Classical H1N1 SIV	CY085113.1	A/swine/Hong Kong/1845/1994(H1N1)
385	Classical H1N1 SIV	MH293472.1	A/swine/Guangdong/1/2007(H1N1)
386	Classical H1N1 SIV	CY085049.2	A/swine/Hong Kong/1219/1993(H1N1)
387	Classical H1N1 SIV	CY084969.1	A/swine/Hong Kong/158/1993(H1N1)
388	Classical H1N1 SIV	CY085017.2	A/swine/Hong Kong/172/1993(H1N1)
389	Classical H1N1 SIV	GQ229277.1	A/swine/Hong Kong/103/1993(H1N1)
390	Classical H1N1 SIV	CY085057.2	A/swine/Hong Kong/1223/1993(H1N1)
391	Classical H1N1 SIV	CY085073.2	A/swine/Hong Kong/1286/1993(H1N1)
392	Classical H1N1 SIV	EU743159.1	A/turkey/IA/21089-3/1992(H1N1)
393	Classical H1N1 SIV	CY084985.1	A/swine/Hong Kong/299/1993(H1N1)
394	Classical H1N1 SIV	CY085065.1	A/swine/Hong Kong/835/1993(H1N1)
395	Classical H1N1 SIV	CY085033.2	A/swine/Hong Kong/813/1993(H1N1)
396	Classical H1N1 SIV	CY085041.2	A/swine/Hong Kong/829/1993(H1N1)
397	Classical H1N1 SIV	CY084993.2	A/swine/Hong Kong/347/1993(H1N1)
398	Classical H1N1 SIV	CY085089.1	A/swine/Hong Kong/1374/1993(H1N1)
399	Classical H1N1 SIV	CY085001.1	A/swine/Hong Kong/574/1993(H1N1)
400	Classical H1N1 SIV	U46020.1	A/swine/Hong Kong/172/1993(H1N1)
401	Classical H1N1 SIV	GU052267.1	A/Swine/Indiana/1726/1988(H1N1)
402	Classical H1N1 SIV	CY086317.1	A/swine/Hong Kong/8372/2001(H1N1)
403	Classical H1N1 SIV	CY086037.1	A/swine/Hong Kong/4026/1999(H1N1)
404	Classical H1N1 SIV	CY086213.1	A/swine/Hong Kong/5279/1999(H1N1)
405	Classical H1N1 SIV	CY086199.1	A/swine/Hong Kong/5269/1999(H1N1)

406	Classical H1N1 SIV	CY086321.1	A/swine/Hong Kong/8596/2001(H1N1)
407	Classical H1N1 SIV	CY086349.1	A/swine/Hong Kong/NS896/2001(H1N1)
408	Classical H1N1 SIV	CY086361.1	A/swine/Hong Kong/9314/2001(H1N1)
409	Classical H1N1 SIV	CY086345.1	A/swine/Hong Kong/NS884/2001(H1N1)
410	Classical H1N1 SIV	CY086347.1	A/swine/Hong Kong/NS888/2001(H1N1)
411	Classical H1N1 SIV	CY085382.2	A/swine/Hong Kong/8376/2001(H1N1)
412	Classical H1N1 SIV	CY085462.2	A/swine/Hong Kong/NS1586/2001(H1N1)
413	Classical H1N1 SIV	CY085414.2	A/swine/Hong Kong/NS885/2001(H1N1)
414	Classical H1N1 SIV	CY085310.2	A/swine/Hong Kong/6301/2000(H1N1)
415	Classical H1N1 SIV	CY085398.2	A/swine/Hong Kong/8690/2001(H1N1)
416	Classical H1N1 SIV	CY085390.2	A/swine/Hong Kong/8631/2001(H1N1)
417	Classical H1N1 SIV	CY085358.2	A/swine/Hong Kong/NS241/2000(H1N1)
418	Classical H1N1 SIV	CY085366.2	A/swine/Hong Kong/7945/2000(H1N1)
419	Classical H1N1 SIV	CY085334.2	A/swine/Hong Kong/NS9/2000(H1N1)
420	Classical H1N1 SIV	CY085342.2	A/swine/Hong Kong/7635/2000(H1N1)
421	Classical H1N1 SIV	CY086309.1	A/swine/Hong Kong/NS246/2000(H1N1)
422	Classical H1N1 SIV	CY086307.1	A/swine/Hong Kong/NS217/2000(H1N1)
423	H3N2 SIV	EPI505732	A/swine/China/JG20/2019
424	H3N2 SIV	EPI18968262	A/swine/Guangxi/JG20/2019
425	H3N2 SIV	EPI18968292	A/swine/Guangxi/JGKP/2020
426	H3N2 SIV	EPI256395	A/swine/Jiangsu/POS3593/2016
427	H3N2 SIV	EPI103839	A/swine/Hong Kong/NS2439/2011
428	H3N2 SIV	EPI103838	A/swine/Guangxi/NS2783/2010

429	H3N2 SIV	EPI103840	A/swine/Hong Kong/2503/2011
430	H3N2 SIV	EPI165152	A/swine/Guangxi/NNXD/2013
431	H3N2 SIV	EPI165153	A/swine/Guangxi/JGB4/2013
432	H3N2 SIV	EPI282063	A/swine/Guangxi/JG1/2014
433	H3N2 SIV	EPI20075804	A/swine/Shandong/ZYZ/2021
434	H3N2 SIV	EPI6795400	A/swine/Shandong/15/2018
435	H3N2 SIV	EPI19616456	A/swine/China/ZQ82/2018
436	H3N2 SIV	EPI19616455	A/swine/China/F9-3/2018
437	H3N2 SIV	EPI256394	A/swine/Jiangsu/P3589/2016
438	H3N2 SIV	EPI20075800	A/swine/Shandong/1224C/2020
439	H3N2 SIV	EPI20075887	A/swine/Shandong/S21/2020
440	H3N2 SIV	EPI20076501	A/swine/Shandong/1224D/2020
441	H3N2 SIV	EPI505731	A/swine/China/JG13/2019
442	H3N2 SIV	EPI18968263	A/swine/Guangxi/JG13/2019
443	H3N2 SIV	EPI19616454	A/swine/China/1720/2017
444	H3N2 SIV	EPI18968307	A/swine/Guangxi/NNZB222/2022
445	H3N2 SIV	EPI18968310	A/swine/Guangxi/NNMS41/2023
446	H3N2 SIV	EPI148107	A/swine/Guangdong/L22/2010
447	H3N2 SIV	EPI81518	A/swine/Guangdong/7/2006
448	H3N2 SIV	EPI146762	A/swine/Guangdong/L5/2010
449	H3N2 SIV	EPI22865	A/swine/Sichuan/01/2006
450	H3N2 SIV	EPI138597	A/swine/HuNan/01/2008
451	H3N2 SIV	EPI30073	A/swine/Guangdong/Z5/2003

452	H3N2 SIV	EPI143743	A/swine/Henan/1/2010
453	H3N2 SIV	EPI4657	A/swine/Guangdong/6/2004
454	H3N2 SIV	EPI4653	A/swine/Guangdong/1/2003
455	H3N2 SIV	EPI13899	A/swine/Guangdong/166/06
456	H3N2 SIV	EPI29268	A/swine/Guangdong/03/2005
457	H3N2 SIV	EPI65628	A/swine/Guangxi/1/2004
458	H3N2 SIV	EPI13897	A/swine/Guangdong/164/06
459	H3N2 SIV	EPI13898	A/swine/Guangdong/165/06
460	H3N2 SIV	EPI29269	A/swine/Guangdong/04/2005
461	H3N2 SIV	EPI4655	A/swine/Guangdong/4/2003
462	H3N2 SIV	EPI4656	A/swine/Guangdong/5/2003
463	H3N2 SIV	EPI141390	A/swine/Eastern China/S17/2003
464	H3N2 SIV	EPI4654	A/swine/Guangdong/3/2003
465	H3N2 SIV	EPI11406	A/swine/Henan/S4/2001
466	H3N2 SIV	EPI33554	A/swine/Hebei/1/2005
467	H3N2 SIV	EPI81519	A/swine/Fujian/43/2007
468	H3N2 SIV	EPI81520	A/swine/Shandong/106/2007
469	H3N2 SIV	EPI81521	A/swine/Shandong/133/2007
470	H3N2 SIV	EPI29267	A/swine/Guangdong/02/2005
471	H3N2 SIV	EPI63613	A/swine/Jilin/5/2007
472	H3N2 SIV	EPI63612	A/swine/Jilin/19/2007
473	H3N2 SIV	EPI63179	A/swine/Jilin/37/2008
474	H3N2 SIV	EPI77606	A/swine/Heilongjiang/10/2007

475	H3N2 SIV	EPI18968298	A/swine/Guangxi/JGX3/2020
476	H3N2 SIV	EPI124039	A/swine/Hunan/3/2008
477	H3N2 SIV	EPI148108	A/swine/Guangdong/L23/2010
478	H3N2 SIV	EPI67906	A/swine/Guangdong/106/2002
479	H3N2 SIV	EPI67909	A/swine/Guangdong/111/2002
480	H3N2 SIV	EPI81523	A/swine/Guangdong/223/2006
481	H3N2 SIV	EPI64592	A/swine/Guangdong/102/2002
482	H3N2 SIV	EPI67907	A/swine/Guangdong/107/2002
483	H3N2 SIV	EPI67908	A/swine/Guangdong/110/2002
484	H3N2 SIV	EPI67910	A/swine/Guangdong/113/2002
485	H3N2 SIV	EPI81525	A/swine/Guangdong/811/2006
486	H3N2 SIV	EPI81522	A/swine/Guangdong/211/2006
487	H3N2 SIV	EPI81524	A/swine/Guangdong/423/2006
488	H3N2 SIV	EPI69960	A/swine/Guangdong/SGD6
489	H3N2 SIV	EPI8475	A/swine/Heilongjiang/74/2000
490	H3N2 SIV	EPI91632	A/swine/Fujian/F2/2007
491	H3N2 SIV	EPI13896	A/swine/Heilongjiang/1/05
492	H3N2 SIV	EPI29506	A/swine/Guangdong/01/2002
493	H3N2 SIV	EPI81526	A/swine/Guangdong/968/2006
494	H3N2 SIV	EPI127500	A/swine/Guangdong/L21/2011
495	H3N2 SIV	EPI8474	A/swine/Inner Mongolia/547/2001
496	H3N2 SIV	EPI4664	A/swine/Fujian/668/01
497	H5N6 SIV	EPI266596	A/swine/Guangdong/G3/2015

498	H5N6 SIV	EPI196100	A/swine/Guangdong/1/2014
499	H5N6 SIV	EPI196101	A/swine/Guangdong/2/2014
500	H5N6 SIV	EPI14174523	A/swine/China/RZ/2018
501	H5N1 SIV	EPI4579	A/swine/Fujian/1/2003
502	H5N1 SIV	EPI226073	A/swine/Shandong/SD1/2014
503	H5N1 SIV	EPI4580	A/swine/Fujian/F1/2001
504	H5N1 SIV	EPI68082	A/swine/Fujian/2001
505	H5N1 SIV	EPI67419	A/swine/Fujian/2003
506	H5N1 SIV	EPI65326	A/swine/Henan/wy/2004
507	H5N1 SIV	EPI65327	A/swine/Guangxi/wz/2004
508	H5N1 SIV	EPI226074	A/swine/Shandong/SD2/2014
509	H5N1 SIV	EPI65330	A/swine/Anhui/ca/2004
510	H5N1 SIV	EPI4138	A/swine/Shandong/2/03
511	H5N1 SIV	EPI65323	A/swine/Anhui/cb/2004
512	H5N1 SIV	EPI144532	A/swine/Jiangsu/2/2009
513	H5N1 SIV	EPI144531	A/swine/Jiangsu/1/2008
514	H5N1 SIV	EPI17767115	A/swine/Guangxi/592/2011
515	H5N1 SIV	EPI17732887	A/swine/Guangxi/592/2011
516	H5N1 SIV	EPI393329	A/swine/Zhejiang/SW57/2015
517	H6N6 SIV	EPI19167974	A/swine/Chile/CPN3853/2023
518	H6N6 SIV	EPI139117	A/swine/Yangzhou/080/2009
519	H6N6 SIV	EPI89164	A/swine/Guangdong/K6/2010
520	H9N2 SIV	EPI503942	A/swine/Shandong/TA009/2019

521	H9N2 SIV	EPI381335	A/swine/China/SPF embryonated chicken eggs/2015
522	H9N2 SIV	EPI84439	A/swine/Taizhou/5/2008
523	H9N2 SIV	EPI84440	A/swine/Yangzhou/1/2008
524	H9N2 SIV	EPI139173	A/swine/Shanghai/Y1/2009
525	H9N2 SIV	EPI139172	A/swine/Henan/Y1/2009
526	H9N2 SIV	EPI19139971	A/swine/China/def2/2011
527	H9N2 SIV	EPI81609	A/swine/Guangxi/8/2007
528	H9N2 SIV	EPI81608	A/swine/Guangxi/7/2007
529	H9N2 SIV	EPI81611	A/swine/Guangxi/10/2007
530	H9N2 SIV	EPI81610	A/swine/Guangxi/9/2007
531	H9N2 SIV	EPI10587	A/swine/Henan/3/2004
532	H9N2 SIV	EPI10593	A/swine/Henan/2/2004
533	H9N2 SIV	EPI10588	A/swine/Henan/4/2004
534	H9N2 SIV	EPI10594	A/swine/Henan/6/2004
535	H9N2 SIV	EPI10590	A/swine/Henan/5/2004
536	H9N2 SIV	EPI10589	A/swine/Henan/7/2004
537	H9N2 SIV	EPI10591	A/swine/Henan/8/2004
538	H9N2 SIV	EPI65625	A/swine/Guangdong/wxl/2004
539	H9N2 SIV	EPI15583	A/swine/Jiangxi/1/2004
540	H9N2 SIV	EPI15584	A/swine/Jiangxi/wx2/2004
541	H9N2 SIV	EPI67424	A/swine/Shandong/w4/2003
542	H9N2 SIV	EPI84311	A/swine/Shandong/3/2003
543	H9N2 SIV	EPI84312	A/swine/Shandong/8/2003

544	H9N2 SIV	EPI67429	A/swine/Shandong/fNY/2003
545	H9N2 SIV	EPI67428	A/swine/Shandong/fLS/2003
546	H9N2 SIV	EPI67430	A/swine/Shandong/fZC/2003
547	H9N2 SIV	EPI67427	A/swine/Shandong/fJN/2003
548	H9N2 SIV	EPI67426	A/swine/Shandong/fHZ/2003
549	H9N2 SIV	EPI13020	A/swine/Guangxi/FS2/2005
550	H9N2 SIV	EPI13022	A/swine/Guangxi/S15/2005
551	H9N2 SIV	EPI13023	A/swine/Guangxi/S11/2005
552	H9N2 SIV	EPI12580	A/swine/Guangxi/58/2005
553	H9N2 SIV	EPI179451	A/swine/Guangdong/L1/2010
554	H9N2 SIV	EPI64951	A/swine/Shandong/nc/2005
555	H9N2 SIV	EPI67423	A/swine/Shandong/nb/2003
556	H9N2 SIV	EPI67422	A/swine/Shandong/na/2003
557	H9N2 SIV	EPI75419	A/swine/Hebei/012/2008
558	H9N2 SIV	EPI229212	A/swine/Yantai/16/2012
